# Anti-Neuroinflammatory and Neuroprotective Effect of Intermedin B Isolated from the *Curcuma longa* L. via NF-κB and ROS Inhibition in BV2 Microglia and HT22 Hippocampal Cells

**DOI:** 10.3390/ijms24087390

**Published:** 2023-04-17

**Authors:** Hwan Lee, Zhiming Liu, Linsha Dong, Dae Young Lee, Dahye Yoon, Hyuncheol Oh, Youn-Chul Kim, Ren-Bo An, Dong-Sung Lee

**Affiliations:** 1 College of Pharmacy, Chosun University, Gwangju 61452, Republic of Korea; ghksdldi123@hanmail.net (H.L.); lzmqust@126.com (Z.L.); donglinsha011@163.com (L.D.); 2 Department of Herbal Crop Research, National Institute of Horticultural and Herbal Science, Rural Development Administration (RDA), Eumseong 27709, Republic of Koreadahyeyoon@korea.kr (D.Y.); 3 College of Pharmacy, Wonkwang University, Iksan 54538, Republic of Korea; hoh@wonkwang.ac.kr (H.O.); yckim@wku.ac.kr (Y.-C.K.); 4 College of Pharmacy, Yanbian University, Yanji 133002, China

**Keywords:** *Curcuma longa* L., neurodegenerative disease, intermedin B, BV2 microglia, HT22 hippocampus

## Abstract

Compounds derived from *Curcuma longa* L. (*C. longa*) have been extensively studied and reported to be effective and safe for the prevention and treatment of various diseases, but most research has been focused on curcuminoids derived from *C. longa*. As neurodegenerative diseases are associated with oxidation and inflammation, the present study aimed to isolate and identify active compounds other than curcuminoids from *C. longa* to develop substances to treat these diseases. Seventeen known compounds, including curcuminoids, were chromatographically isolated from the methanol extracts of *C. longa*, and their chemical structures were identified using 1D and 2D NMR spectroscopy. Among the isolated compounds, intermedin B exhibited the best antioxidant effect in the hippocampus and anti-inflammatory effect in microglia. Furthermore, intermedin B was confirmed to inhibit the nuclear translocation of NF-κB p-65 and IκBα, exerting anti-inflammatory effects and inhibiting the generation of reactive oxygen species, exerting neuroprotective effects. These results highlight the research value of active components other than curcuminoids in *C. longa*-derived compounds and suggest that intermedin B may be a promising candidate for the prevention of neurodegenerative diseases.

## 1. Introduction

As our society ages, research interests have been shifting to anti-aging modes of action. Diseases associated with aging generally refer to conditions that arise from physiological changes that occur as a person ages. Such diseases include chronic ailments and neurological disorders, and result from a decline in the body’s physiological functions. This can lead to an imbalance in the immune system, resulting in the development of related diseases [1].

Aging of the immune system can cause inflammation by interfering with the production of immune cells and increasing pro-inflammatory cytokines [2]. Microglia, which are found in the central nervous system (CNS), play critical roles in inflammation and immunity. They help remove foreign substances caused by toxins, protect nerve cells, and maintain homeostasis [3]. However, when activated excessively by various factors, they secrete nitric oxide (NO) and pro-inflammatory mediators such as cytokines, prostaglandin E2 (PGE2), tumor necrosis factor-α (TNF-α), interleukin (IL)-6, and reactive oxygen species (ROS), leading to inflammation in the nervous system [4,5]. The production and release of inflammatory substances can lead to cellular and brain damage, ultimately resulting in neurodegenerative diseases. Therefore, the production of NO and PGE2 is induced by the pro-inflammatory proteins inducible nitric oxide synthase (iNOS) and cyclooxygenase-2 (COX-2), and the regulation of inflammatory mediators including pro-inflammatory proteins is considered an important target for treating neuroinflammation.

NF-κB exists in an inactive state bound to IκB in the cytoplasm, but upon stimulation, it dissociates from IκB, allowing the subunit p65 to translocate to the nucleus and activate the transcription of various cell proliferation and inflammation-related genes, including iNOS, COX-2, and TNF-α [6]. B-cell inhibitor alpha (IκBα) is a factor that controls the activation of NF-κB by preventing its translocation into the nucleus within the cell. However, when IκBα is phosphorylated, its ability to bind with NF-κB decreases, allowing the NF-κB subunits p50 and p65 to translocate into the nucleus and become activated [7]. Phosphorylated IκBα is called p-IκBα, and unlike IκBα, it can translocate into the nucleus and play a critical role in the activation of NF-κB. To study NF-κB activation, another method is to inhibit the nuclear translocation of p65, which is a critical protein in the NF-κB signaling pathway. When NF-κB is activated, p65 forms a complex with p50 and translocates to the nucleus to regulate diverse biological responses, including inflammatory and immune responses [8].

Activated neurons are tissues characterized by high levels of oxygen consumption and the strong generation of ROS. While ROS are normally suppressed by the antioxidant network, excessive ROS can cause neuronal damage and impair cognitive function, leading to age-related diseases that promote oxidative stress, including not only neurodegenerative diseases but also trauma and ischemic injuries [9,10]. HT22 neuronal cells have been used as a valuable model for studying the mechanisms underlying glutamate-induced oxidative stress leading to cell death. HT22 hippocampal cells do not experience toxicity through receptors due to the lack of ionotropic glutamate receptors (iGluRs), and cysteine uptake is inhibited through the glutamate/cystine antiporter when exposed to glutamate [11]. As a result, the decrease in intracellular levels of glutathione, a precursor of cysteine, and the increase in ROS production and Ca^2+^ levels induce oxidative stress [12,13]. Therefore, regulating ROS production in hippocampal cells is considered an important target for verifying the efficacy of interventions aimed at reducing cellular damage caused by oxidative stress [14].

Neurodegenerative diseases, such as Alzheimer’s disease (AD), Parkinson’s disease (PD), and Huntington’s disease (HD), are caused by the dysfunction and loss of nerve cells in the CNS. Oxidative stress is a major factor contributing to the development of these diseases, and chronic inflammation is closely related to it, as ROS and inflammation exacerbate each other in a vicious cycle [15]. One of the triggers for the formation of ROS and reactive nitrogen species (RNS) is elevated levels of redox-active transition metals in the brain, including copper, iron, and zinc, which can lead to necrotic neuronal cell death [16]. In the brains of patients with AD, copper ions have been reported to be associated with forming free radicals, and overactivated microglia cannot maintain iron homeostasis, resulting in the release of pro-inflammatory cytokines and free radicals due to excess iron [17,18]. Therefore, it is crucial to identify compounds that can inhibit oxidative stress and inflammation caused by metal chelation to suppress age-related diseases.

*Curcuma longa* L. (*C. longa*) is an herb belonging to the ginger family and is native to South Asia. It is widely used as a spice and natural cosmetic in Indian, Iranian, and Thai cuisine, and has been used in Ayurveda and Oriental medicine for the prevention and treatment of various ailments, including dermatosis and depression [19]. *C. longa* has also been traditionally used as a treatment for gastrointestinal problems, colds, and headaches [20]. It has also been used as a treatment for various diseases such as skin inflammation, arthritis, and cancer [21,22]. *C. longa* has been extensively studied for the beneficial effects of its extracts and compounds, and has been reported to be effective and safe for the prevention and treatment of several diseases [23,24]. However, most studies on *C. longa* compounds are related to curcuminoids, and reports on other compounds are rare. Therefore, the purpose of this study was to isolate various compounds from *C. longa* and investigate their anti-inflammatory and neuroprotective effects in BV2 microglia and HT22 hippocampal cells to confirm the research value of *C. longa*-derived compounds other than curcuminoids. In addition, we aimed to discover candidate compounds derived *C. longa* other than curcumin for the prevention of neurodegenerative diseases.

## 2. Results

### 2.1. Chemical Structures of 17 Compounds Isolated from C. longa

**Figure 1:**
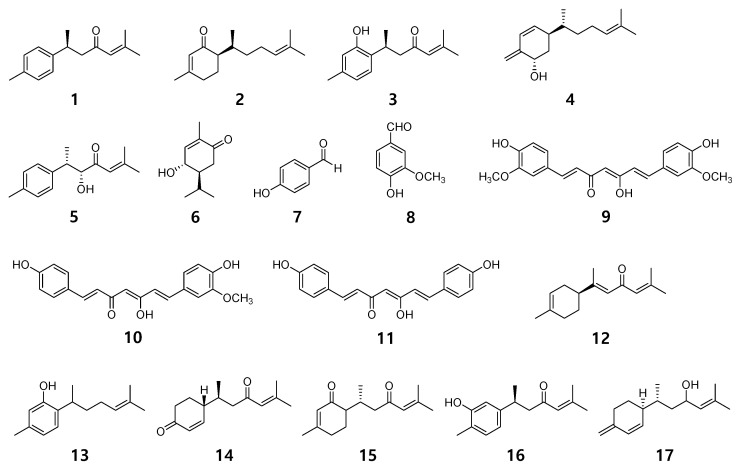
Chemical structures of compounds **1**–**17** from *C. longa*.

Seventeen compounds were isolated from the methanol extract of *C. longa* using various chromatographic methods. It was confirmed that the isolated compounds were structurally (+)-(S)-ar-turmerone (**1**) [25], (6R)-6-[(S)-1, 5-dimethylhex-4-en-1-yl]-3-methylcyclo-hex-2-en-1-one (**2**) [26,27], turmeronol B (**3**) [27,28], intermedin B (**4**) [29], 8-hydroxy-ar-turmerone (**5**) [30], 5-hydroxy-2-oxo-p-menth-6(1)-ene (**6**) [31,32], 4-hydroxybenzaldehyde (**7**) [33], vanillic aldehyde (**8**) [34], curcumin (**9**) [35], demethoxycurcumin (**10**) [35,36], bisdemethoxycurcumin (**11**) [35], (+)-α-atlantone (**12**) [37], curcuphenol (**13**) [38,39], 4-(1′,5′-dimethyl-3′-oxo-4′-hexenyl)-2-cyclohexen-1-one (**14**) [40,41], bisabolone-9-one (**15**) [30,42], turmeronol A (**16**) [28], and bisacurol (**17**) [43,44], by comparing the nuclear magnetic resonance spectroscopy data analysis results with the analysis results reported in the reference literature (Figure 1, Figures S1–S17).

### 2.2. Effects of 17 Compounds from C. longa on Nitrite Inhibition in LPS-Induced BV2 Microglia

**Figure 2:**
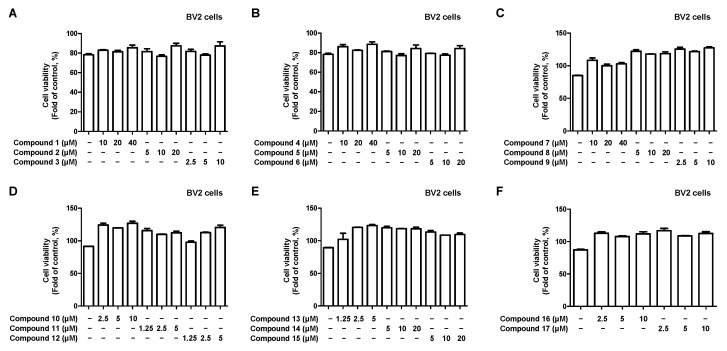
Effect of 17 natural compounds isolated from *C. longa* on cytotoxicity in BV2 microglia. (**A**–**F**) Cytotoxicity was evaluated in cells treated for 48 h with 1.25 to 40 μM of compounds **1**–**17**. Data are presented as the mean ± SD values of 3 independent experiments.

We investigated the anti-neuroinflammatory effects of 17 compounds isolated from *C. longa* on lipopolysaccharide (LPS)-induced BV2 microglia. First, we performed a cytotoxicity evaluation to determine the treatment concentrations of each compound. No toxicity was observed at concentrations of 5 μM for compounds **11**, **12**, and **13**; 10 μM for compounds **3**, **9**, **10**, **16**, and **17**; 20 μM for compounds **2**, **5**, **6**, **8**, **14**, and **15**; and 40 μM for compounds **1**, **4**, and **7** (Figure 2).

**Figure 3:**
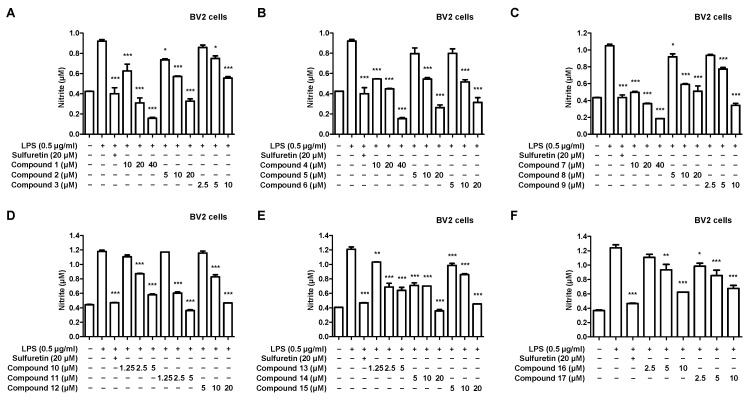
Effect of 17 natural compounds isolated from *C. longa* on inhibiting nitrite production in LPS-induced BV2 microglia. (**A**–**F**) Microglia were treated with each concentration of the compound and cultured for 8 h, followed by treatment with LPS. After LPS treatment, the cells were cultured for 18 h, and the supernatant was collected to investigate the effect of nitrite inhibition. The positive control group was treated with sulfuretin 20 µM. Data are presented as the mean ± SD values of 3 independent experiments. * *p* < 0.05, ** *p* < 0.01, and *** *p* < 0.001 compared with LPS. Sulfuretin used as a positive control.

Next, we investigated the nitrite inhibitory effect using LPS-induced BV2 microglia, with the individual treatment concentration of the compound set based on the results of the cytotoxicity evaluation. All 17 compounds isolated from *C. longa* inhibited nitrite in a concentration-dependent manner (Figure 3).

### 2.3. Effects of 17 Compounds from C. longa on Oxidative Stress in Glutamate-Induced HT22 Hippocampal Cells

**Figure 4:**
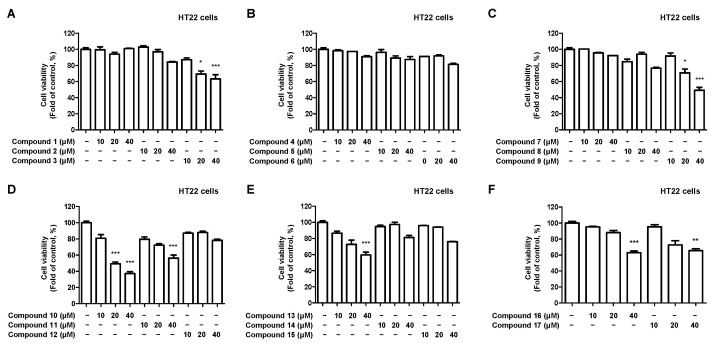
Effect of the 17 natural compounds isolated from *C. longa* on the cytotoxicity in HT22 hippocampal cells. (**A**–**F**) Cytotoxicity was evaluated in cells treated for 48 h with 10–40 μM of compounds 1–17. Data are presented as the mean ± SD values of 3 independent experiments. * *p* < 0.05, ** *p* < 0.01, and *** *p* < 0.001 compared with control.

We investigated the neuroprotective effects of 17 compounds isolated from *C. longa* against oxidative stress in glutamate-induced HT22 hippocampal cells. Firstly, a cytotoxicity evaluation was performed to determine the treatment concentration of the compound. Toxicity was observed at 20 μM for compounds **3**, **9**, **10**, and 40 μM for compounds **11**, **13**, **16**, and **17** (Figure 4).

**Figure 5:**
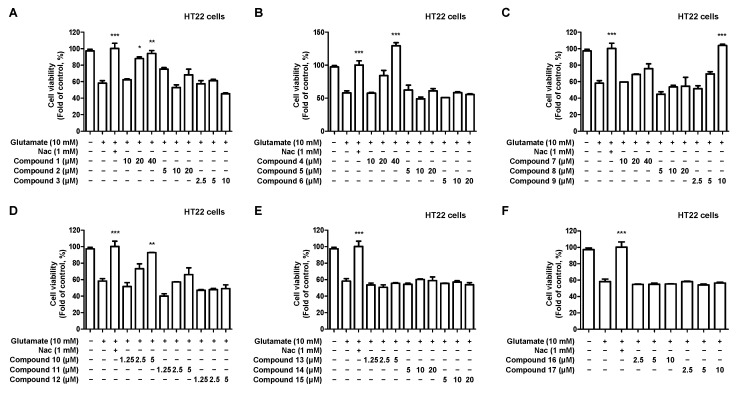
Effect of 17 natural compounds isolated from *C. longa* on the neuroprotection in glutamate-induced HT22 hippocampal cells. (**A**–**F**) HT22 hippocampal cells were treated with each concentration of the compound and cultured for 8 h, followed by treatment with glutamate. After glutamate treatment, the cells were cultured for 12 h, and an MTT assay was performed to determine the neuroprotective effect. The positive control group was treated with N-acetyl cysteine (Nac) 1 mM. Data are presented as the mean ± SD values of 3 independent experiments; * *p* < 0.05, ** *p* < 0.01, and *** *p* < 0.001 compared with glutamate.

Next, the neuroprotective effects of the compounds were investigated in glutamate-induced HT22 hippocampal cells. The individual treatment concentrations of each compound were determined based on the results of the cytotoxicity evaluation. Compounds **1**, **4**, **9**, and **10** showed concentration-dependent neuroprotective effects, with compounds **4** and **9** exhibiting the strongest effects (Figure 5).

### 2.4. Effects of Intermedin B on Levels of Pro-Inflammatory Mediators and Cytokines in LPS-Induced BV2 Microglia

**Figure 6:**
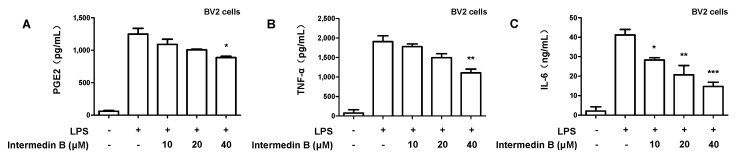
Effect of intermedin B (**4**) isolated from *C. longa* on inhibiting PGE2 (**A**), TNF-α (**B**), and IL-6 (**C**) production in LPS-induced BV2 microglia. Microglia were treated with each concentration of intermedin B (**4**) and cultured for 8 h, followed by treatment with LPS. After LPS treatment, the cells were cultured for 18 h, and the supernatant was collected to investigate the effect of PGE2, TNF-α, and IL-6 inhibition. Data are presented as the mean ± SD values of 3 independent experiments. * *p* < 0.05, ** *p* < 0.01, and *** *p* < 0.001 compared with LPS.

Investigation of the nitrite inhibitory effect in LPS-induced BV2 microglia and neuroprotective effects in glutamate-induced HT22 hippocampal cells revealed that intermedin B (**4**) and curcumin (**9**) exhibited the highest NO inhibitory and neuroprotective effects. However, numerous studies have reported the anti-inflammatory and antioxidant effects of curcumin (**9**) [45,46,47,48]. In this regard, intermedin B (**4**) was administered to LPS-induced BV2 microglia to investigate its inhibitory effect on the production of PGE2, an inflammatory cytokine. Intermedin B (**4**) suppressed the production of PGE2 in a concentration-dependent manner (Figure 6A). Subsequently, the inhibitory effect of intermedin B (**4**) on TNF-α and IL-6 production was examined. The results indicated that intermedin B (**4**) inhibited the production of TNF-α and IL-6 in a concentration-dependent manner (Figure 6B,C). These findings demonstrated that intermedin B (**4**) suppressed the production of inflammatory cytokines in LPS-induced BV2 microglia.

**Figure 7:**
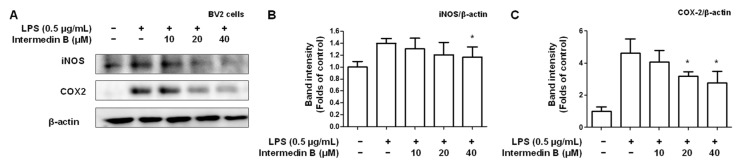
Effect of intermedin B (**4**) isolated from *C. longa* on inhibiting iNOS and COX-2 expression in LPS-induced BV2 microglia. To examine the inhibitory effect of intermedin B (**4**) on the expression of pro-inflammatory proteins iNOS and COX-2, cells were treated with LPS for 18 h following pretreatment with 10–40 μM of intermedin B (**4**). (**A**) Western blotting was performed, and the immunoblot was quantified using ImageJ software to quantify the expression levels of iNOS (**B**) and COX-2 (**C**). The intensities of the bands were normalized to those of *β*-actin, and the data are presented as the mean ± SD values of 3 independent experiments. * *p* < 0.05 compared with LPS.

Subsequently, the inhibitory effects of intermedin B (**4**) on the expression of inflammatory cytokines related to iNOS and COX-2 were investigated. As a result, intermedin B (**4**) was found to inhibit the expression levels of iNOS and COX-2 in a concentration-dependent manner (Figure 7).

### 2.5. Effects of Intermedin B on NF-κB Activation in LPS-Induced BV2 Microglia

**Figure 8:**
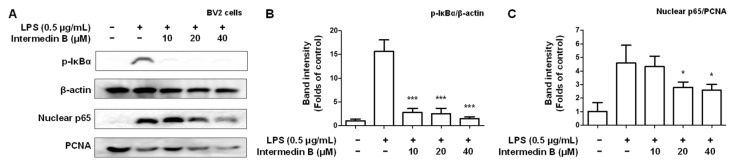
Effect of intermedin B (**4**) isolated from *C. longa* on inhibiting nuclear factor-kappa B (NF-κB) p65 activation in LPS-induced BV2 microglia. To examine the inhibitory effect of intermedin B (**4**) on the activation of NF-κB p65 and p-IκBα, cells were treated with LPS for 0.5 h following pretreatment with 10–40 μM of intermedin B (**4**). (**A**) Western blotting was performed, and the immunoblot was quantified using ImageJ software to quantify the expression levels of p-IκBα (**B**) and p65 (**C**). The intensities of the bands were normalized to those of *β*-actin and PCNA, and the data are presented as mean ± SD of 3 independent experiments. * *p* < 0.05, and *** *p* < 0.001 compared with LPS.

We investigated whether intermedin B (**4**) was involved in regulating NF-κB in LPS-induced BV2 microglia. To investigate whether intermedin B (**4**) was involved in NF-κB control, cytosolic and nuclear extracts were extracted from LPS-induced BV2 microglia. As a result, intermedin B (**4**) was inhibited by the nuclear factor degradation of the kappa light polypeptide gene enhancer and nuclear translocation of p-IκBα and p65 in a concentration-dependent manner (Figure 8).

### 2.6. Effect of Intermedin B on Glutamate-Induced ROS Generation in HT22 Hippocampal Cells

**Figure 9:**
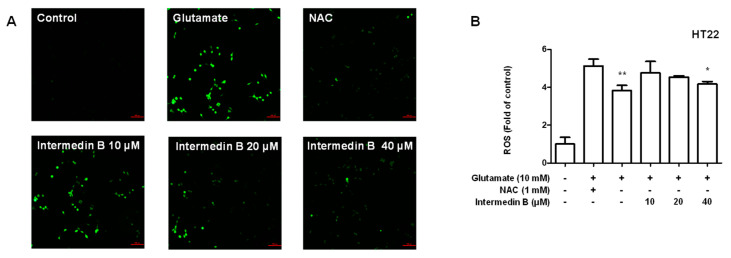
Effect of intermedin B (**4**) isolated from *C. longa* on inhibiting ROS production in glutamate-induced HT22 hippocampal cells. (**A**) HT22 hippocampal cells were pre-treated with intermedin B (**4**) for 3 h, followed by exposure to 10 mM glutamate for 8 h. The cells were then loaded with 10 μM 2′-7′dichlorofluorescin diacetate (DCFH-DA) and measured using a fluorescence microscope. (**B**) Fluorescence intensities were quantified using ImageJ software. The positive control group was treated with N-acetyl cysteine (Nac) 1 mM. Data are presented as the mean ± SD values of 3 independent experiments. * *p* < 0.05, and ** *p* < 0.01 compared with glutamate.

We investigated whether intermedin B (**4**) showed an inhibitory effect on ROS generation based on its results in suppressing oxidative stress in glutamate-induced HT22 hippocampal cells. These results confirm that intermedin B (**4**) inhibited ROS generation in a concentration-dependent manner (Figure 9).

## 3. Discussion

Curcumin, the main chemical component of *C. longa*, has antioxidant and anti-inflammatory effects, and *C. longa* has attracted a lot of scientific interest in both traditional and modern medicine [49,50,51]. Recently, studies on the anti-cancer effects of *C. longa* have been conducted, and evidence has shown that *C. longa* helps prevent and treat various types of cancer [52,53,54]. Although there have been various studies on *C. longa* extracts and curcuminoids, few studies have been reported on the other *C. longa*-derived compounds, even though there are numerous preventive and therapeutic effects of *C. longa* extracts and curcuminoids [55,56]. Therefore, we attempted to identify potential candidates for preventing and treating neurodegenerative diseases using *C. longa*-derived compounds other than curcuminoids. Firstly, we isolated 17 compounds from the methanol extract of *C. longa* by column chromatography.

Anti-inflammatory and antioxidant strategies are important for preventing and treating neurodegenerative diseases. Nitrite is a biologically active nitrogen oxide that acts as an anti-inflammatory agent in the body. It acts as an intermediate of the NO generated in the digestive tract and is transported to various tissues through the bloodstream. NO is produced in vascular endothelial cells and plays an important role in regulating inflammatory responses. In addition, NO reduces inflammation by inhibiting the production of inflammatory chemicals and suppressing the inflammatory response in vascular endothelial cells [57]. However, suppose inflammation occurs due to various causes and the concentration of NO becomes abnormally high; in that case, it combines with other molecules in cells, leading to powerful oxidative effects and oxidative stress [58]. Oxidative stress has been reported to contribute to the development and progression of inflammatory diseases [59,60]. We investigated the nitrite inhibitory effects of the isolated compounds using LPS-induced BV2 microliga (Figure 3). All isolated compounds showed concentration-dependent inhibitory effects on nitrite production. Next, we confirmed the neuroprotective effects of the isolated compounds on oxidative stress in glutamate-induced HT22 hippocampal cells (Figure 5). Compounds **1**, **4**, **9**, and **10** showed concentration-dependent neuroprotective effects against oxidative stress, and particularly compounds **4** and **9** showed the most potent neuroprotective effects. Based on these results, anti-inflammatory and antioxidant studies were further conducted using compound **4**, excluding curcumin, as the objective of this study.

Microglia are cells belonging to the body’s immune system and play a role in inflammation. PGE2, TNF-α, and IL-6 are inflammatory cytokines that play a role in inducing inflammatory responses. These cytokines are produced in microglia, causing an increase in inflammatory conditions. PGE2 plays an important role in developing pain due to inflammation, whereas TNF-α promotes the inflammatory process [61]. Additionally, IL-6 plays an important role in the immune response, but its excessive production is associated with inflammatory diseases [62]. Therefore, the interaction between mast cells and inflammatory cytokines plays an important role in the occurrence and development of inflammatory diseases. Based on this, it was found that intermedin B (**4**) inhibited the excessive production of the inflammatory cytokines PGE2, TNF-α, and IL-6 in inflammatory responses in a concentration-dependent manner (Figure 6). Subsequently, the expression of iNOS and COX-2, enzymes involved in the synthesis of inflammatory mediators such as NO and PGE2, was investigated. As a result, it was confirmed that intermedin B (**4**) inhibited the protein expression levels of iNOS and COX-2 (Figure 7). It is, therefore, confirmed that intermedin B (**4**) isolated from *C. longa* effectively regulates the expression of inflammatory mediators and pro-inflammatory proteins.

Noxious inflammatory agents and cytokines, including NO, PGE2, TNF-α, IL-6, iNOS, and COX-2, are regulated by transcription factors such as NF-κB, a key signaling molecule that activates various genes involved in inflammation control. Therefore, inhibition of NF-κB activation by the phosphorylation and degradation of IκBα and translocation of the NF-κB p65/p50 heterodimer to the nucleus could be an effective therapeutic strategy for inflammatory diseases. We conducted a study to investigate whether the process of intermedin B (**4**) inhibiting inflammatory mediators and pro-inflammatory proteins by intermedin B (**4**) was associated with the activation of NF-κB (Figure 8). As a result, it was found that intermedin B (**4**) inhibited the nuclear translocation of IκBα and p65 induced by LPS.

ROS, also known as active oxygen, are highly activated oxygen molecules generated inside and outside the cell. Some ROS are generated within the cell, such as in the mitochondrial respiratory process and NADPH oxidase in the endoplasmic reticulum. Additionally, ROS can be produced outside of the cell by environmental pollution, sunlight, cigarette smoke, and other factors. ROS play an important role in the oxidative metabolism of cells. However, excessive ROS production can lead to oxidative stress, damaging cellular components such as DNA, proteins, and lipids, resulting in aging, mutation, cell death, and other outcomes. Oxidative stress is also associated with aging and brain disorders. In particular, an increase in ROS levels has been observed in diseases such as AD, PD, and vascular dementia, which are age-related and degenerative brain disorders. This is because oxidative stress within brain tissue is linked to brain cell damage and cell death. Therefore, methods to inhibit ROS and oxidative stress are being researched as preventive and therapeutic strategies for degenerative brain disorders. Based on this theory, we investigated whether intermedin B (**4**) inhibited ROS production in glutamate-induced HT22 hippocampal cells (Figure 9). The results confirmed that intermedin B (**4**) inhibited ROS production in a concentration-dependent manner.

Based on these results, it was confirmed that intermedin B (**4**) inhibits the translocation of p-IκBα and the activation of NF-κB in BV2 microglia, thereby suppressing the expression of iNOS and COX-2, which are pro-inflammatory proteins and inhibiting the production of inflammatory cytokines. Furthermore, it was shown to inhibit the generation of ROS in HT22 hippocampal cells, thereby protecting neurons and preventing neuronal damage and cognitive impairment. These findings suggest that intermedin B (**4**) is effective in preventing and treating neurodegenerative diseases in vitro. This study also revealed the significant activity of other compounds, apart from curcuminoids, among compounds isolated from *C. longa*. In addition, further studies investigating the use of intermedin B (**4**) on the in vivo neuroprotective effect and neuronal structure are needed.

## 4. Materials and Methods

### 4.1. Extraction and Isolation Materials

*C. longa* was harvested from the Jindo-country, Jeonnam-province, Korea. The plant was identified by comparing with a specimen (voucher No. MPS004295) from the National Institute of Horticultural and Herbal Science (NIHHS), Eumseong, Korea. Reagent-grade solvent was used for extraction and column chromatography (CC). CC was performed using YMC ODS-A (C18) (YMC, Kyoto, Japan), Cytiva Sephadex™ LH-20 (Cytiva, Marlborough, MA, USA), and silica gel (Merck, Darmstadt, Germany). Subsequently, 1D and 2D NMR spectra were recorded in chloroform-d, acetone-d6 and dimethyl sulfoxide (DMSO)-d6 using a JEOL JNM ECP-400 spectrometer (400 MHz ^1^H and 100 MHz ^13^C).

### 4.2. Plant Material and Extraction

The extract was then added at 80 °C for 3 h, after adding 6 L of methanol to 0.7 kg of *C. longa* to obtain 64.4 g of extract. Subsequently, 64.4 g methanol extract was suspended in 1 L distilled water (11.9 g), and *n*-hexane (18.3 g), CH_2_Cl_2_ (20.2 g), EtOAC (8.8 g), and *n*-BuOH (1.9 g) were sequentially added; subsequently, the solvent fraction was obtained.

The *n*-hexane fraction (18.3 g) was isolated by silica gel CC under hexane:EtOAc (50:1–1:2) conditions to obtain 19 fractions (HX-1–HX-19). HX-2 (5.56 g) was isolated using ODS-A C-18 under MeOH:water (3:1) conditions and divided into three sub-fractions to obtain compound **12** (HX-2-3, 2.9 mg). HX-3 (217.4 mg) was isolated using ODS-A C-18 under MeOH:water (3:1) conditions and divided into three sub-fractions to obtain compound **1** (HX-3-2, 8.9 mg). HX3-3 (8.4 mg) was purified by silica gel CC under hexane:EtOAc (50:1) conditions to obtain compound **2** (HX-3-3-1, 1.1 mg) and compound **13** (HX-3-3-2, 3.8 mg). HX-4 (131.3 mg) was isolated using ODS-A C-18 in MeOH:water (2:1) and divided into two sub-fractions to obtain compound **2** (HX-4-2, 43.7 mg). HX-5 (108.9 mg) was isolated using ODS-A C-18 under MeOH:water (2:1) conditions and divided into five sub-fractions to obtain compound **5** (HX-5-1, 11.3 mg). HX-6 (310.3 mg) was isolated using ODS-A C-18 under MeOH:water (3:1) conditions and divided into five sub-fractions to obtain compound **5** (HX-6-1, 31.3 mg). HX-7 (197.9 mg) was isolated using ODS-A C-18 under MeOH:water (3:1) conditions and divided into five sub-fractions to obtain compound **3** (HX-7-1, 7.7 mg). HX-8 (321.1 mg) was isolated by ODS-A C-18 under MeOH:water (2:3) conditions and divided into four sub-fractions to obtain compounds **3** (HX-8-1, 6.0 mg) and **17** (HX-8-2, 4.9 mg). HX-10 (118.0 mg) was isolated into ODS-A C-18 under MeOH:water (6:4) conditions and divided into five sub-fractions to obtain compounds **14** (HX-10-1, 2.1 mg), **15** (HX -10-2, 10.4 mg), and **16** (HX-10-3, 20.7 mg).

The CH_2_Cl_2_ fraction (16.8 g) was isolated by silica gel CC under hexane:EtOAc (30:1–3:1) conditions to obtain 12 fractions (DM-1–DM-16). DM-4 (418.8 mg) was isolated using ODS-A C-18 under MeOH:water (3:2–2:1) conditions and divided into five sub-fractions to obtain compound **1** (DM-4-3, 156.7 mg). DM-6 (40.0 mg) was isolated using ODS-A C-18 under MeOH:water (2:1) conditions and divided into three sub-fractions to obtain compound **2** (DM-6-3, 2.1 mg). DM-7 (34.0 mg) was isolated by ODS-A C-18 under MeOH:water (3:2) conditions and divided into two subfractions to obtain compound **5** (DM-7-1, 7.8 mg). DM-9 (169.4 mg) was isolated by ODS-A C-18 under MeOH:water (1:1–3:1) conditions and divided into five subfractions to obtain compound **3** (DM-9-2, 14.3 mg). DM-11 (529.7 mg) was isolated using ODS-A C-18 under MeOH:water (1:1–7:1) conditions to obtain 12 fractions (DM-11-1–DM-11-12). DM-11-1 (31.9 mg) was isolated by silica gel CC under CH_2_Cl_2_:MeOH (80:1) conditions and divided into two sub-fractions to obtain compound **7** (DM-11-1, 9.4 mg) and compound **8** (DM-11-2, 17.6 mg). DM-11-5 (12.1 mg) was isolated by ODS-A C-18 under MeOH:water (3:2) conditions and divided into two subfractions to obtain compound **6** (DM-11-5-1, 2.8 mg). DM-11-12 (206.7 mg) was isolated by silica gel CC under CH_2_Cl_2_:MeOH (50:1) conditions and divided into five sub-fractions to obtain compound **4** (DM-11-12-2, 38.5 mg). DM-14 (3.45 g) was isolated using ODS-A C-18 in MeOH:water (3:1) to obtain seven fractions (DM-14-1–DM-14-7). DM-14-7 (163.0 mg) was isolated by ODS-A C-18 under MeOH:water (5:1–7:1) conditions and divided into two sub-fractions to obtain compounds **9** (DM-14-7-1, 2.6 mg) and **10** (DM-14-7-2, 19.0 mg).

The EtOAc fraction (8.8 g) was isolated using silica gel CC in CH_2_Cl_2_:MeOH:water (100:1:0–7:3:0.3) to obtain seven fractions (EA-1–EA-7). EA-4 (1.0 g) was isolated by silica gel CC under CH_2_Cl_2_:MeOH (50:1) conditions to obtain 12 fractions (EA-4-1–EA-4-12). EA-4-9 (348.8 mg) was purified by sepadex LH-20 CC under CH_2_Cl_2_:Hexane:MeOH (5:5:1) conditions to obtain compound **11** (EA-4-9, 322.7 mg).

(+)-(S)-ar-turmerone (**1**): ^1^H NMR (400 MHz, Chloroform-d6); δ 7.10 (4H, brs, ArH), 6.02 (1H, m, H-10), 3.29 (1H, m, H-7), 2.71 (1H, dd, *J* = 15.7, 6.0 Hz, H-8), 2.61 (1H, dd, *J* = 15.7, 8.3 Hz, H-8), 2.31 (3H, s, H-15), 2.11 (3H, s, H-13), 1.85 (3H, d, *J* = 1.0 Hz, H-12), 1.24 (3H, d, *J* = 6.9 Hz, H-14). ^13^C NMR (100 MHz, Chloroform-d6); δ 199.9 (C-9), 155.1 (C-11), 143.8 (C-1), 135.6 (C-4), 129.2 (C-3,C-5), 126.8 (C-2,C-6), 124.2 (C-10), 52.8 (C-8), 35.4 (C-7), 27.7 (C-13), 22.1 (C-14), 21.1 (C-15), 20.8 (C-12).

(6R)-6-[(S)-1,5-dimethylhex-4-en-1-yl]-3-methylcyclo-hex-2-en-1-one (**2**): ^1^H NMR (400 MHz, Chloroform-d6); δ 5.85 (1H, d, *J* = 1.2 Hz, H-2), 5.10 (1H, t, *J* = 7.0 Hz, H-10), 1.92 (3H, s, H-15), 1.67 (3H, s, H-12), 1.58 (3H, s, H-13), 0.79 (3H, d, *J* = 6.8 Hz, H-14). ^13^C NMR (100 MHz, Chloroform-d6); δ 201.2 (C-1), 161.2 (C-3), 131.5 (C-11), 127.3 (C-2), 124.6 (C-10), 49.9 (C-6), 34.8 (C-8), 31.0 (C-4), 30.4 (C-7), 26.1 (C-9), 25.8 (C-12), 24.2 (C-15), 22.5 (C-5), 17.7 (C-13), 15.7 (C-14).

Turmeronol B (**3**): ^1^H NMR (400 MHz, Chloroform-d6); δ 8.07 (1H, s, 2-OH), 7.02 (1H, dd, *J* = 7.8, 1.2 Hz, H-6), 6.73 (1H, s, H-3), 6.70 (1H, d, *J* = 8.0 Hz, H-5), 6.00 (1H, s, H-10), 3.56 (1H, m, H-7), 2.79 (2H, m, H-8), 2.25 (3H, s, H-15), 2.11 (3H, s, H-13), 1.85 (3H, s, H-12), 1.28 (3H, dd, *J* = 7.1, 1.7 Hz, H-14). ^13^C NMR (100 MHz, Chloroform-d6); δ 202.0 (C-9), 158.0 (C-2), 153.8 (C-11), 137.2 (C-4), 130.3 (C-1), 126.1 (C-6), 123.2 (C-10), 121.7 (C-5), 118.5 (C-3), 54.2 (C-8), 28.0 (C-12), 25.8 (C-7), 21.5 (C-14), 21.2 (C-15), 21.0 (C-13).

Intermedin B (**4**): ^1^H NMR (400 MHz, Chloroform-d6); δ 6.12 (1H, dd, *J* = 10.0, 2.6 Hz, H-2), 6.04 (1H, s, H-10), 5.73 (1H, d, *J* = 10.0 Hz, H-3), 5.03 (1H, s, H-15b), 4.93 (1H, s, H-15a), 4.40 (1H, br s, H-5); 2.11 (3H, s, CH3-13), 1.85 (3H, s, CH3-12), 0.87 (3H, d, *J* = 6.2 Hz, CH3-14). ^13^C NMR (100 MHz, Chloroform-d6); δ 200.7 (C-9), 155.4 (C-11), 144.9 (C-4), 133.7 (C-2), 127.2 (C-3), 124.1 (C-10), 113.6 (C-15), 69.3 (C-5), 48.7 (C-8), 35.8 (C-7), 32.9 (C-1), 31.8 (C-6), 27.7 (C-13), 20.8 (C-12), 16.7 (C-14).

8-hydroxy-ar-turmerone (**5**): ^1^H NMR (400 MHz, Chloroform-d6); δ 7.24 (2H, d, *J* = 7.9 Hz, H-2, 6), 7.13 (2H, d, *J* = 7.9 Hz, H-3, 5), 6.10 (1H, m, H-10), 4.29 (1H, m, H-8), 3.15 (1H, m, H-7), 2.32 (3H, s, H-15), 2.21 (3H, s, H-13), 1.94 (3H, s, H-12), 1.11 (3H, d, *J* = 7.1 Hz, H-14). ^13^C NMR (100 MHz, Chloroform-d6); δ 200.2 (C-9), 159.7 (C-11), 140.9 (C-4), 136.2 (C-1), 129.1 (C-2, 6), 127.7 (C-3, 5), 119.8 (C-10), 80.5 (C-8), 42.4 (C-7), 28.1 (C-12), 21.5 (C-15), 21.1 (C-13), 14.0 (C-14).

5-hydroxy-2-oxo-p-menth-6(1)-ene (**6**): ^1^H NMR (400 MHz, Chloroform-d6); δ 6.64 (1H, m, H-6), 4.34 (1H, m, H-5), 1.77 (3H, dd, *J* = 2.0, 1.4 Hz, H-7), 0.96 (3H, d, *J* = 6.8 Hz, H-10), 0.88 (3H, d, *J* = 6.9 Hz, H-9). ^13^C NMR (100 MHz, Chloroform-d6); δ 199.9 (C-2), 148.4 (C-6), 135.3 (C-1), 69.2 (C-5), 50.1 (C-4), 36.4 (C-3), 26.4 (C-8), 20.5 (C-10), 16.6 (C-9), 15.3 (C-7).

4-Hydroxybenzaldehyde (**7**): ^1^H NMR (400 MHz, Acetone-d6); δ 9.84 (1H, s, H-7), 7.79 (2H, d, *J* = 8.8 Hz, H-2, 6), 7.00 (2H, d, *J* = 8.8 Hz, H-3,5). ^13^C NMR (100 MHz, Acetone-d6); δ 190.2 (C-7), 163.1 (C-4), 132.0 (C-2, C-6), 129.7 (C-1), 115.8 (C-3, C-5).

Vanillic aldehyde (**8**): ^1^H NMR (400 MHz, Chloroform-d6); δ 9.81 (1H, s, CHO-1), 7.41 (1H, dd, *J* = 8.5, 1.8 Hz, H-6), 7.40 (1H, d, *J* = 1.8 Hz, H-2), 7.02 (1H, d, *J* = 8.5 Hz, H-5), 6.34 (1H, s, 4-HO), 3.94 (3H, s, CH3O-3). ^13^C NMR (100 MHz, Chloroform-d6); δ 191.0 (CHO-1), 151.8 (C-4), 147.2 (C-3), 129.9 (C-1), 127.6 (C-6), 114.5 (C-2), 108.8 (C-5), 56.2 (3-OCH3).

Curcumin (**9**): ^1^H NMR (400 MHz, Acetone-d6); δ 7.59 (2H, d, *J* = 15.8 Hz, H-3, 3′), 7.32 (2H, d, *J* = 2.0 Hz, H-6, 6′), 7.17 (2H, dd, *J* = 8.2, 2.0 Hz, H-10, 10′), 6.88 (2H, d, *J* = 8.2 Hz, H-9, 9′), 6.70 (2H, d, *J* = 15.8 Hz, H-4, 4′), 5.97 (1H, s, H-1), 3.91 (3H, s, 3-OCH3). ^13^C NMR (100 MHz, Acetone-d6); δ 183.7 (C-2, 2′), 149.2 (C-8, 8′), 148.0 (C-7, 7′), 140.6 (C-4, 4′), 127.4 (C-5, 5′), 123.0 (C-10, 10′), 121.5 (C-3, 3′), 115.4 (C-9, 9′), 110.7 (C-6, 6′), 100.8 (C-1), 55.5 (3- OCH3).

Demethoxycurcumin (**10**): ^1^H NMR (400 MHz, Acetone-d6); δ 7.60 (1H, d, *J* = 15.8 Hz, H-3), 7.59 (1H, d, *J* = 15.8 Hz, H-3′), 7.56 (2H, d, *J* = 8.6 Hz, H-7, 9), 7.33 (1H, d, *J* = 1.8 Hz, H-6′), 7.17 (1H, dd, *J* = 8.2, 1.8 Hz, H-10′), 6.88 (3H, t, *J* = 8.2 Hz, H-10, 9′, 6), 6.71 (1H, d, *J* = 15.8 Hz, H-4), 6.65 (1H, d, *J* = 15.8 Hz, H-4′), 5.97 (1H, s, H-1), 3.91 (3H, s, 7′-OCH3). ^13^C NMR (100 MHz, Acetone-d6); δ 183.8 (C-2′), 183.7 (C-2), 159.7 (C-8′), 149.2 (C-8), 148.0 (C-7), 140.6 (C-4), 140.2 (C-4′), 130.2 (C-10′, 6′), 127.4 (C-5), 126.9 (C-5′), 123.1 (C-10), 121.5 (C-3′), 121.3 (C-3), 116.0 (C-9′), 115.4 (C-9), 110.7 (C-6), 100.9 (C-1), 55.5 (7′-OCH3).

Bisdemethoxycurcumin (**11**): ^1^H NMR (400 MHz, DMSO-d6); δ 10.03 (2H, s, OH-8,8′), 7.57 (4H, d, *J* = 8.7 Hz, H-7, 7′, 9, 9′), 7.55 (2H, d, *J* = 16.0 Hz, H-3, 3′), 6.83 (4H, d, *J* = 8.7Hz, H-6, 6′,10, 10′), 6.69 (2H, d, *J* = 16.0 Hz, H-4, 4′), 6.04 (1H, s, H-1). ^13^C NMR (100 MHz, DMSO-d6); δ 183.8 (C-2, 2′), 160.4 (C-8, 8′), 140.9 (C-4, 4′), 130.9 (C-6, 6′), 126.4 (C-5, 5′), 121.4 (C-3, 3′), 116.5 (C-7, 7′), 101.5 (C-1).

(+)-α-Atlantone (**12**): ^1^H NMR (400 MHz, DMSO-d6); δ 6.06 (1H, m, H-10), 6.04 (1H, m, H-8), 5.40 (1H, m, H-3), 2.15 (3H, d, *J* = 1.2 Hz, H-12), 2.14 (3H, d, *J* = 1.2 Hz, H-14), 1.88 (3H, d, *J* = 1.2 Hz, H-13), 1.65 (3H, s, H-15). ^13^C NMR (100 MHz, DMSO-*d_6_*); δ 192.2 (C-9), 161.8 (C-7), 154.1 (C-11), 133.9 (C-4), 126.6 (C-10), 124.4 (C-8), 120.2 (C-3), 44.6 (C-1), 30.5 (C-5), 30.4 (C-2), 27.8 (C-13), 27.5 (C-6), 23.5 (C-15), 20.6 (C-12), 17.6 (C-14).

Curcuphenol (**13**): ^1^H NMR (400 MHz, Chloroform-d6); δ 7.02 (1H, d, *J* = 7.8 Hz, H-6), 6.71 (1H, dd, *J* = 7.8, 1.6 Hz, H-5), 6.58 (1H, d, *J* = 1.6 Hz, H-3), 5.12 (1H, t, *J* = 7.1 Hz, H-10), 2.95 (1H, m, H-7), 2.26 (3H, s, H-15), 1.67 (3H, d, *J* = 1.2 Hz, H-14), 1.53 (3H, d, *J* = 1.2 Hz, H-12), 1.22 (3H, d, *J* = 6.9 Hz, H-15). ^13^C NMR (100 MHz, Chloroform-d6); δ 152.9 (C-2), 136.6 (C-4), 132.1 (C-11), 130.0 (C-1), 126.9 (C-6), 124.7 (C-10), 121.8 (C-5), 116.2 (C-3), 37.3 (C-8), 31.5 (C-7), 26.2 (C-9), 25.8 (C-13), 21.1 (C-14), 20.9 (C-15), 17.6 (C-12).

4-(1′,5′-dimethyl-3′-oxo-4′-hexenyl)-2-cyclohexen-1-one (**14**): ^1^H NMR (400 MHz, Chloroform-d6); δ 6.83 (1H, dt, *J* = 10.2, 2.0 Hz, H-3), 6.06 (1H, m, H-10), 6.02 (1H, ddd, *J* = 10.2, 2.8, 0.9 Hz, H-2), 2.15 (3H, d, *J* = 1.2 Hz, H-13), 1.89 (3H, d, *J* = 1.2 Hz, H-12), 0.92 (3H, d, *J* = 6.7 Hz, H-14). ^13^C NMR (100 MHz, Chloroform-d6); δ 199.8 (C-4), 199.7 (C-9), 156.1 (C-11), 154.3 (C-3), 130.1 (C-2), 123.9 (C-10), 48.3 (C-8), 40.8 (C-1), 37.5 (C-5), 32.8 (C-7), 27.8 (C-12), 24.3 (C-6), 20.9 (C-13), 16.7 (C-14).

Bisabolone-9-one (**15**): ^1^H NMR (400 MHz, Chloroform-d6); δ 6.08 (1H, m, H-10), 5.81 (1H, m, H-3), 2.76 (1H, m, H-7), 2.40 (1H, dd, *J* = 15.1, 6.5 Hz, H-8a), 2.36 (1H, dd, *J* = 15.1, 8.1 Hz, H-8b), 2.14 (1H, m, H-1), 2.11 (1H, d, *J* = 1.2 Hz, H-12), 1.86 (3H, d, *J* = 1.2 Hz, H-13), 1.91 (3H, d, *J* = 1.2 Hz, H-15), 0.86 (3H, d, *J* = 6.8 Hz, H-14). ^13^C NMR (100 MHz, Chloroform-d6); δ 200.7 (C-2), 200.6 (C-9), 161.4 (C-4), 155.4 (C-11), 126.9 (C-3), 123.9 (C-10), 49.6 (C-1), 49.2 (C-8), 30.5 (C-5), 27.9 (C-7), 27.7 (C-12), 24.1 (C-15), 23.3 (C-6), 20.8 (C-13), 16.8 (C-14).

Turmeronol A (**16**): ^1^H NMR (400 MHz, Chloroform-d6); δ 7.01 (1H, d, *J* = 7.6 Hz, H-5), 6.68 (1H, dd, *J* = 7.6, 1.8 Hz, H-6), 6.65 (1H, d, *J* = 1.8 Hz, H-2), 3.24 (1H, m, H-7), 2.10 (1H, d, *J* = 1.2 Hz, H-12), 1.85 (3H, d, *J* = 1.2 Hz, H-13), 2.19 (3H, s, H-15), 1.21 (3H, d, *J* = 6.9 Hz, H-14). ^13^C NMR (100 MHz, Chloroform-d6); δ 200.2 (C-9), 155.6 (C-3), 154.1 (C-11), 146.0 (C-1), 131.0 (C-5), 124.2 (C-10), 121.6 (C-4), 118.8 (C-6), 113.5 (C-2), 52.7 (C-8), 35.4 (C-7), 27.7 (C-13), 20.8 (C-12), 22.0 (C-15), 15.5 (C-14).

Bisacurol (**17**): ^1^H NMR (400 MHz, Chloroform-d6); δ 6.15 (1H, dd, *J* = 10.0, 2.6 Hz, H-2), 5.65 (1H, d, *J* = 10.0 Hz, H-3), 5.12 (1H, dq, *J* = 8.9, 1.4 Hz, H-10), 4.74 (2H, d, *J* = 6.6 Hz, H-15), 4.43 (1H, q, *J* = 8.7 Hz, H-9), 1.73 (3H, d, *J* = 1.4 Hz, H-12), 1.70 (3H, d, *J* = 1.4 Hz, H-13), 0.85 (3H, d, *J* = 6.7 Hz, H-14). ^13^C NMR (100 MHz, Chloroform-d6); δ 143.7 (C-4), 135.7 (C-11), 134.8 (C-2), 129.8 (C-3), 128.3 (C-10), 110.1 (C-15), 67.3 (C-9), 42.2 (C-8), 40.8 (C-1), 33.7 (C-7), 30.4 (C-5), 25.9 (C-12), 24.5 (C-6), 18.3 (C-13), 16.4 (C-14).

### 4.3. Cell Culture

BV2 microglia and HT22 hippocampal cells were provided by Professor Yun-Cheol Kim from Wonkwang University (Iksan, Korea). Cells were cultured in 100 mm dishes at a density of 5 × 10^6^ cells/dish. The culture medium consisted of α-minimum essential medium (for BV2 microglia) and Dulbecco’s modified essential medium (for HT22 hippocampal cells) supplemented with 10% heat-inactivated fetal bovine serum and 1% antibiotic–antimycotic solution (100 U/mL). The cultures were maintained at 37 °C under 5% CO_2_. All cell culture media were purchased from ATCC (Manassas, VA, USA).

### 4.4. MTT Assay

Mitochondrial reductase converts the tetrazolium salt 3-[4,5-dimethylthiazol-2-yl]-2,5-diphenyltetrazolium bromide (MTT) into insoluble formazan crystals. Thus, the effects of the 17 compounds isolated from *C. longa* on cell viability were measured. To measure cell viability, each cell suspension (1 × 10 cells/mL) was treated for 4 h with 5 mg/mL MTT to form formazan. The formed formazan was dissolved in DMSO, and the absorbance was measured at 540 nm using an enzyme-linked immunosorbent assay (ELISA) microplate reader (Molecular Devices, San Jose, CA, USA).

### 4.5. Measurement of NO Generation

The amount of NO produced, a pro-inflammatory substance, was measured using the Griess reaction. The theory behind the Griess reaction is that nitrite (NO2-) combines with sulfonimide to form a diazonium salt, and the formed diazonium salt combines with N-(1-naphthyl)ethylenediamine to form an azo dye. The azo dyes are red, and the higher the amount of NO, the darker the color. Therefore, the effects of 17 compounds isolated from *C. longa* on NO production were measured. The supernatant of each well in which an inflammatory reaction occurred was reacted with the grease reagent at a ratio of 1:1, and absorbance was measured at 570 nm using a microplate reader. As a positive control group, 20uM of sulfuretin, which has been reported to have excellent anti-inflammatory inhibitory effects, was administered [63].

### 4.6. Measurement of Neuroprotective Effects

The measurement of protective effect against glutamate-induced neurotoxicity in HT22 hippocampal cells was conducted as follows: the cells were cultured for 24 h and then treated with compounds isolated from *C. longa* at various concentrations. After 8 h, the cells were treated with 10 mM glutamate and cultured for another 12 h, followed by measurement of cell viability using the MTT assay at a wavelength of 540 nm. N-acetyl cysteine (Nac) at a concentration of 1 mM was used as a positive control.

### 4.7. PGE_2_ Assay

PGE_2_ measurement was performed as previously described using a commercially available kit from R&D Systems (Minneapolis, MN, USA) [64]. Intermedin B (4) isolated from *C. longa* was added at a concentration of 10–40 μM, and the supernatant of each well in which an inflammatory reaction occurred was analyzed at a wavelength of 450 nm.

### 4.8. IL-6 and TNF-α Assay

Methods for measuring IL-6 and TNF-α were performed as previously described using commercially available kits from BioLegend (San Diego, CA, USA) [64]. Intermedin B (4) isolated from *C. longa* was added at a concentration of 10–40 μM, and the supernatant of each well in which an inflammatory reaction occurred was analyzed at a wavelength of 450 nm.

### 4.9. Western Blot Analysis

iNOS, COX-2, nuclear p65, and p-IκBα protein levels were determined by western blot analysis. To perform western blot analysis, inflamed cells were harvested, and pellets were harvested. Subsequently, it was dissolved in a 20 mm Tris-HCl buffer (pH 7.4) containing a protease inhibitor mixture (0.1 mM phenylmethanesulfonyl fluoride, 5 mg/mL aprotinin, 5 mg/mL pepstatin A, and 1 mg/mL chymostatin). Protein concentration was measured using a protein analysis dye reagent concentrate (#5000006; Bio-Rad Laboratories, Hercules, CA, USA), according to the manufacturer’s guidelines. Equal amounts of protein (30 μg) were analyzed by 7.5% and 12% sodium dodecyl sulfate-polyacrylamide gel electrophoresis. Thereafter, the proteins were electrophoretically transferred to Hybond enhanced chemiluminescence (ECL) nitrocellulose membranes (Bio-Rad Laboratories). The membranes were blocked with 5% skim milk and sequentially incubated with the relevant primary antibody and horseradish peroxidase-conjugated secondary antibody. Finally, proteins were visualized using ECL (Pierce Biotechnology, Rockford, IL, USA).

### 4.10. Preparation of Nucleus and Cytosolic Fraction

For the nuclear extraction method, the nuclear and cytosolic fractions were separated using the Cayman Nuclear Extraction Kit (Cayman, Ann Arbor, MI, USA). Nuclear fraction separation was performed according to the manufacturer’s instructions.

### 4.11. ROS Assays

HT22 hippocampal cells were cultured in 6-well plates (1 × 10^5^ cells/mL) and pre-treated with different concentrations of intermedin B (**4**) for 3 h. The cells were induced with glutamate (5 mM) for 8 h, the medium was removed, and the cells were loaded with 10 μM 2′-7′dichlorofluorescin diacetate (DCFH-DA) in phosphate-buffered saline (PBS). The plates were then incubated at 37 °C for 20 min. After washing with PBS, the images were obtained using a fluorescence microscope (Nikon Ti-S Eclipse; Melville, NY, USA).

### 4.12. Statistical Analysis

Statistical analysis was conducted using GraphPad Software Inc. (San Diego, CA, USA) and GraphPad Prism software version 3.03. The mean difference was determined using a one-way analysis of variance and Newman–Keuls post hoc test, with statistical significance set at *p* < 0.05. All data were obtained from three independent experiments and are presented as mean ± standard deviation.

## 5. Conclusions

In this study, 17 compounds were isolated from *C. longa* to identify the active components other than curcuminoids. Among the isolated compounds, intermedin B (**4**) demonstrated the most significant antioxidant effect in the hippocampus and anti-inflammatory effect in BV2 microglia. Intermedin B (**4**) exhibited anti-inflammatory effects by inhibiting the nuclear translocation of NF-κB p-65 and IκBα, and demonstrated neuroprotective effects by inhibiting ROS generation. The antioxidant and anti-inflammatory effects of intermedin B (**4**) were demonstrated for the first time in vitro, and may provide evidence for its clinical application in the prevention of neurodegenerative diseases.

## Data Availability

The data presented in this study are available in this article. Other data supporting the findings of this study are available upon request from the corresponding authors.

## Supplementary Materials

The following supporting information can be downloaded at: https://www.mdpi.com/article/10.3390/ijms24087390/s1.
