# Supplementary material for: Anti-Neuroinflammatory and Neuroprotective Effect of Intermedin B Isolated from the Curcuma longa L. via NF-κB and ROS Inhibition in BV2 Microglia and HT22 Hippocampal Cells
Source: Int J Mol Sci. 2023 Apr 17;24(8):7390. doi: 10.3390/ijms24087390 (PMC10138482; doi:10.3390/ijms24087390)
Supplement: Supplementary file 1 [file ijms-24-07390-s001.zip › ijms-2328288-supplementary.pdf]

Supplementary Information

Anti-Neuroinflammatory and Neuroprotective Effect of Inter-medin B Isolated from the *Curcuma longa* L. via NF-κB and ROS Inhibition in BV2 microglia and HT22 hippocampus

Hwan Lee [1], Zhiming Liu [1], Linsha Dong [1], Dae Young Lee [2], Dahye Yoon [2], Hyuncheol Oh [3], Youn-Chul Kim [3], Ren-Bo An [4,*] and Dong-Sung Lee [1,*]

[1] College of Pharmacy, Chosun University, Gwangju 61452, Republic of Korea; ghksdldi123@hanmail.net (H.L.); lzmqust@126.com (Z.L.); donglinsha011@163.com (L.D.)
[2] Department of Herbal Crop Research, National Institute of Horticultural and Herbal Science, Rural Development Administration (RDA), Eumseong 27709, Republic of Korea; dylee0809@gmail.com (D.Y.L.); dahyeyoon@korea.kr (D.Y.)
[3] College of Pharmacy, Wonkwang University, Iksan 54538, Republic of Korea; hoh@wonkwang.ac.kr (H.O.); yckim@wku.ac.kr (Y.-C.K.)
[4] College of Pharmacy, Yanbian University, Yanji 133002, China
* Correspondence: anrb@ybu.edu.cn (R.-B.A.); dslee2771@chosun.ac.kr (D.-S.L.)

Contents

Figure S1. ^{1}H- and ^{13}C- NMR spectrum of compound **1**.

Figure S2. ^{1}H- and ^{13}C- NMR spectrum of compound **2**.

Figure S3. ^{1}H- and ^{13}C- NMR spectrum of compound **3**.

Figure S4. ^{1}H- and ^{13}C- NMR spectrum of compound **4**.

Figure S5. ^{1}H- and ^{13}C- NMR spectrum of compound **5**.

Figure S6. ^{1}H- and ^{13}C- NMR spectrum of compound **6**.

Figure S7. ^{1}H- and ^{13}C- NMR spectrum of compound **7**.

Figure S8. ^{1}H- and ^{13}C- NMR spectrum of compound **8**.

Figure S9. ^{1}H- and ^{13}C- NMR spectrum of compound **9**.

Figure S10. ^{1}H- and ^{13}C- NMR spectrum of compound **10**.

Figure S11. ^{1}H- and ^{13}C- NMR spectrum of compound **11**.

Figure S12. ^{1}H- and ^{13}C- NMR spectrum of compound **12**.

Figure S13. ^{1}H- and ^{13}C- NMR spectrum of compound **13**.

Figure S14. ^{1}H- and ^{13}C- NMR spectrum of compound **14**.

Figure S15. ^{1}H- and ^{13}C- NMR spectrum of compound **15**.

Figure S16. ^{1}H- and ^{13}C- NMR spectrum of compound **16**.

Figure S17. ^{1}H- and ^{13}C- NMR spectrum of compound **17**.

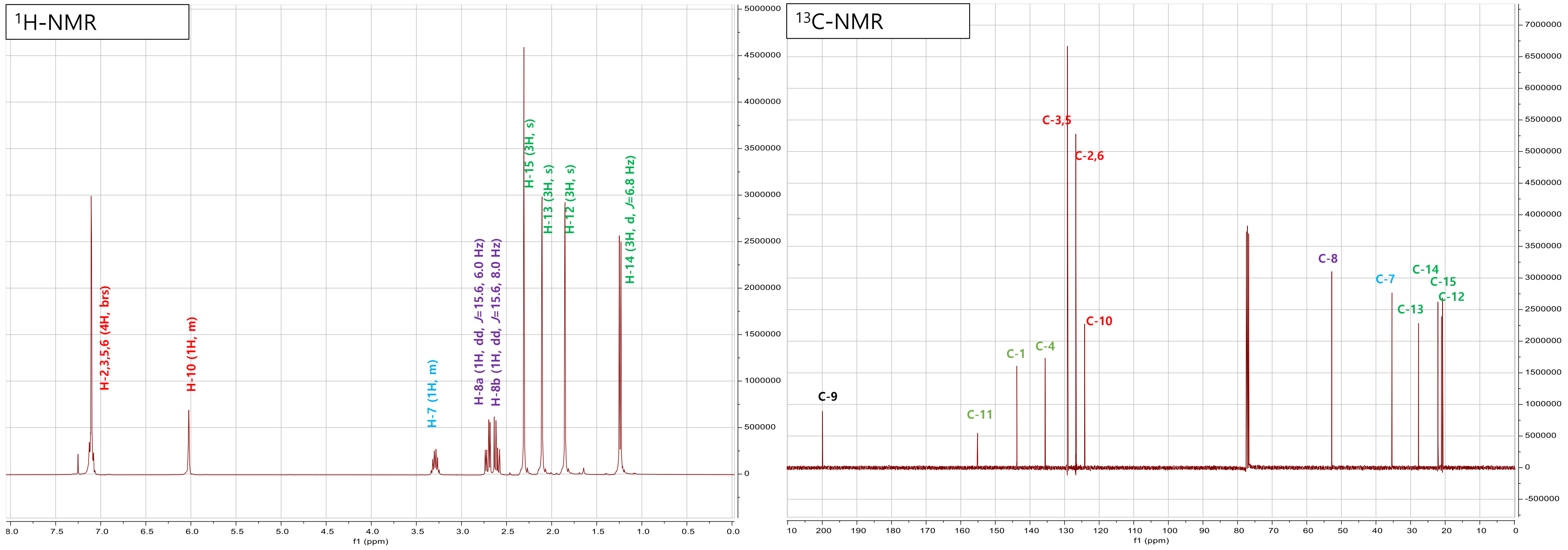

Figure S1. ^{1}H- and ^{13}C- NMR spectrum of compound **1**.

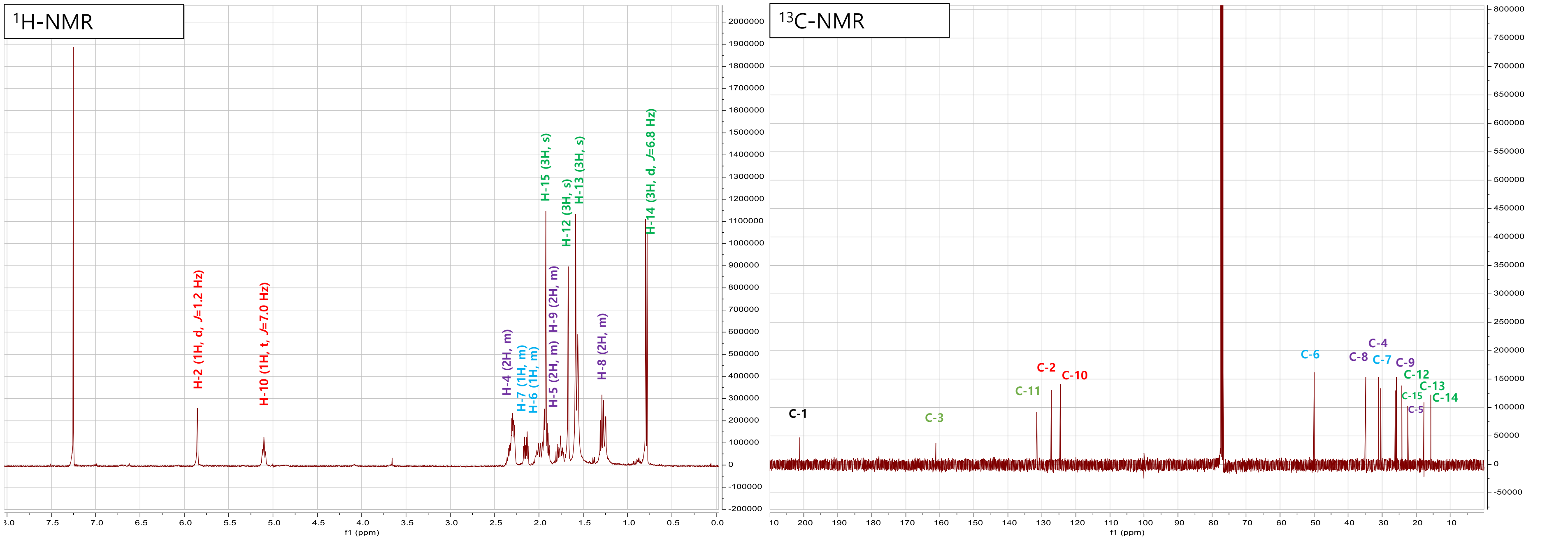

Figure S2. ^{1}H- and ^{13}C- NMR spectrum of compound **2**.

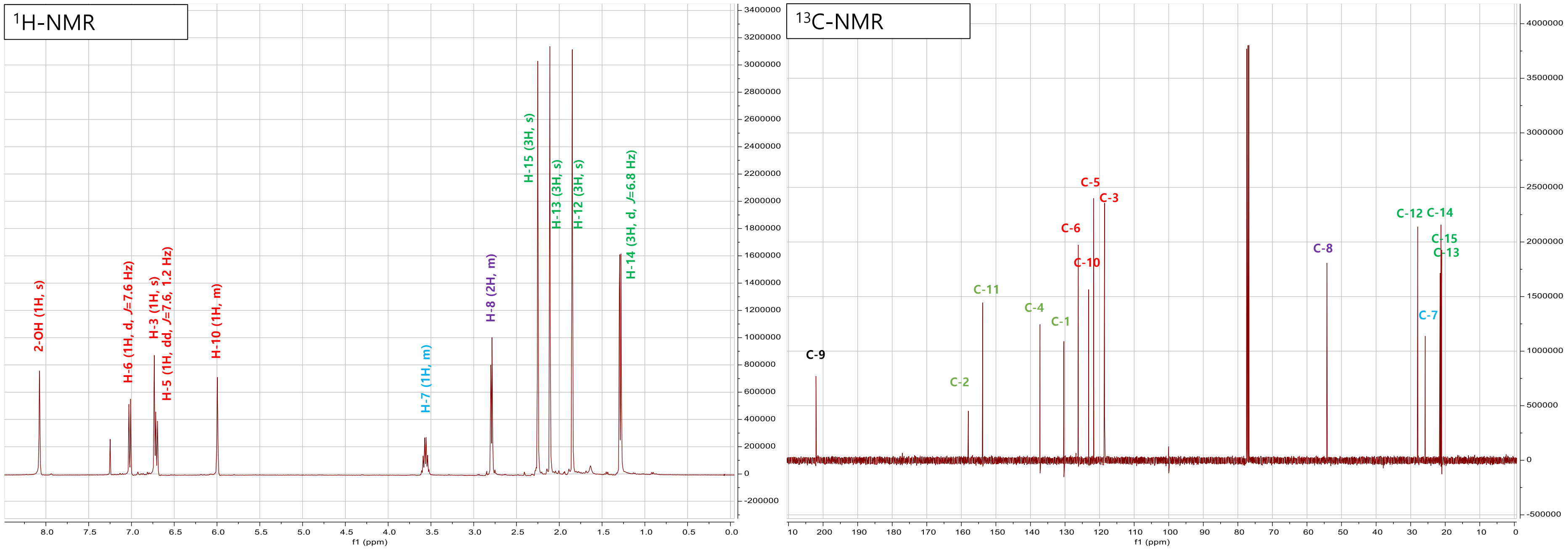

Figure S3. ^{1}H- and ^{13}C- NMR spectrum of compound **3**.

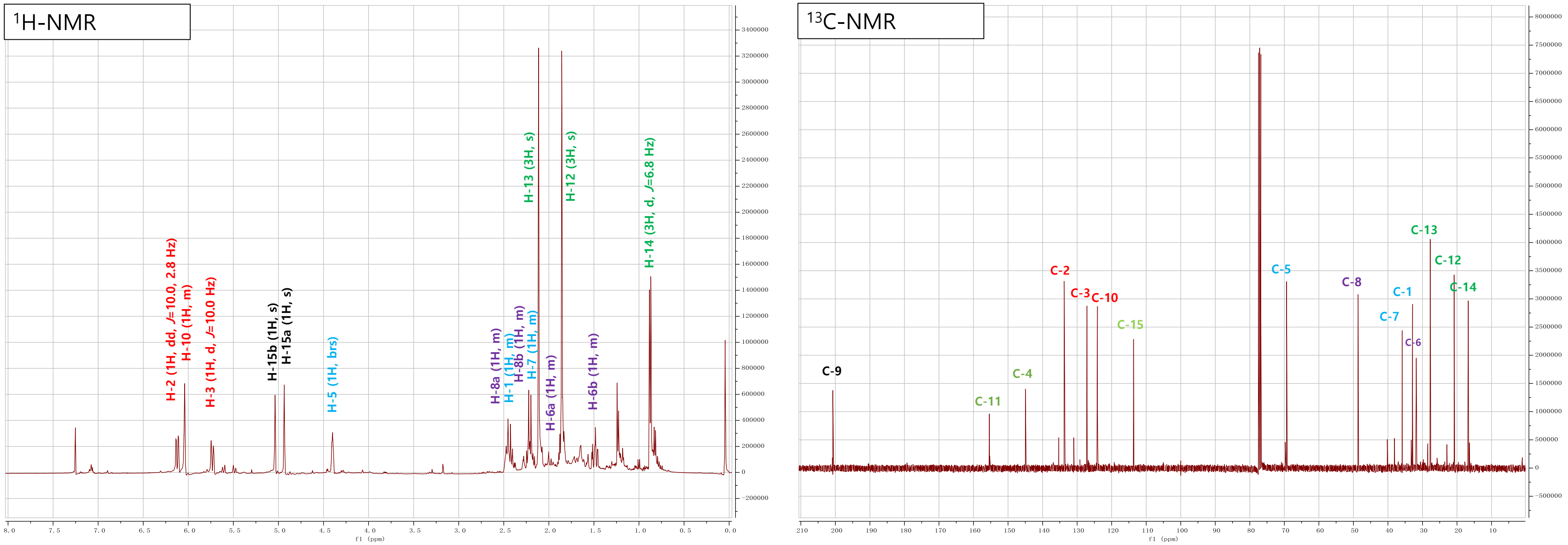

Figure S4. ^{1}H- and ^{13}C- NMR spectrum of compound **4**.

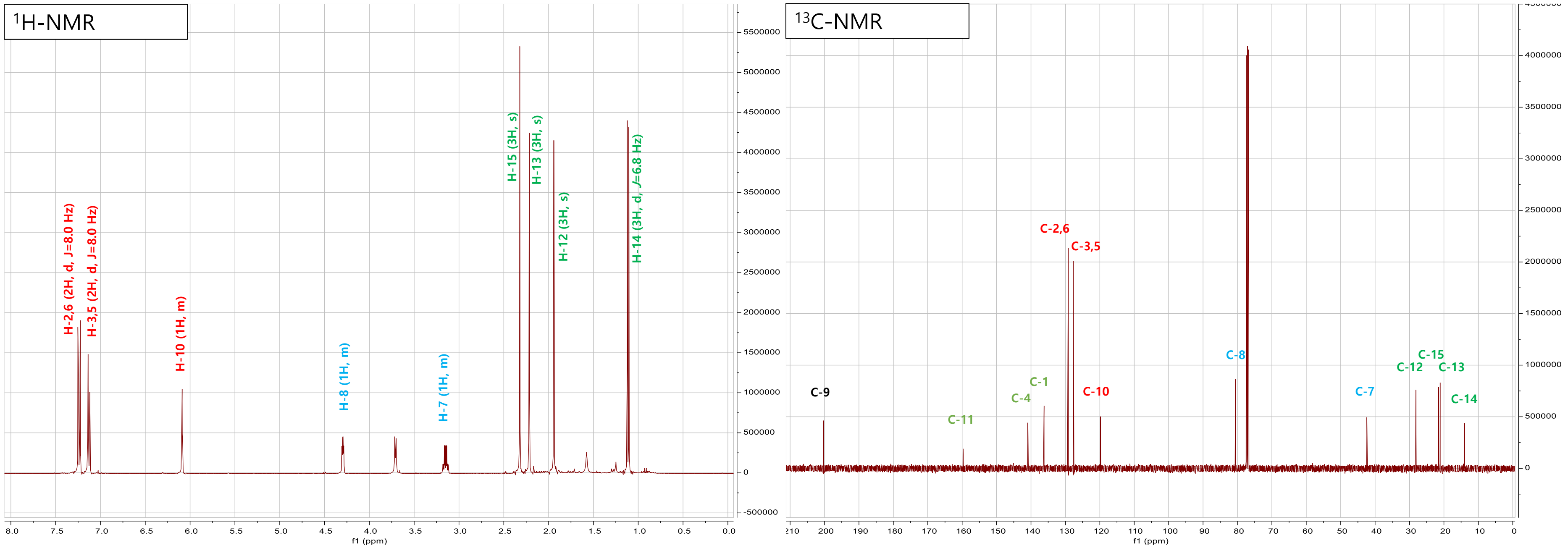

Figure S5. ^{1}H- and ^{13}C- NMR spectrum of compound **5**.

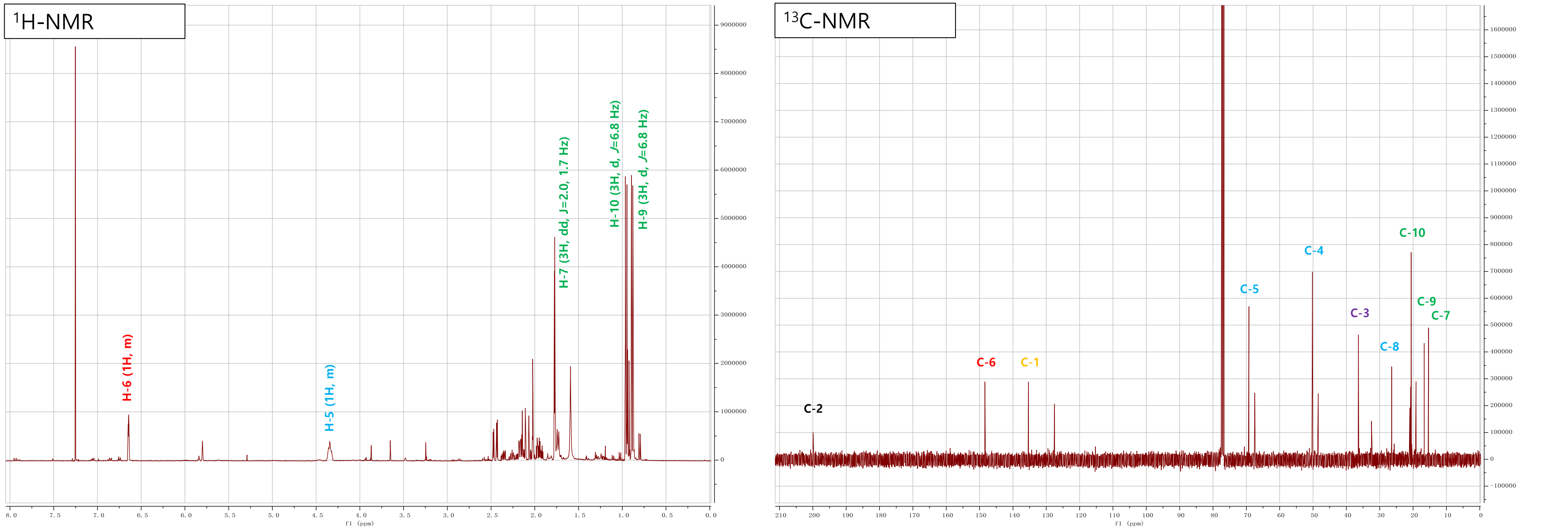

Figure S6. ^{1}H- and ^{13}C- NMR spectrum of compound **6**.

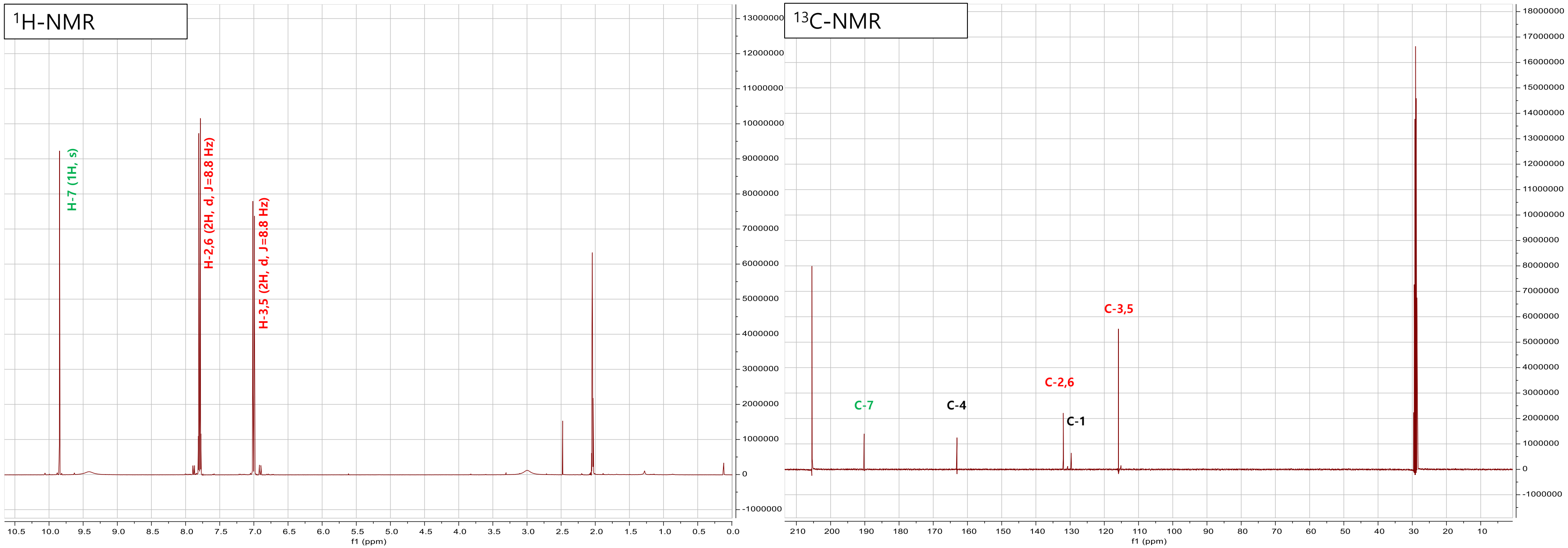

Figure S7. ^{1}H- and ^{13}C- NMR spectrum of compound **7**.

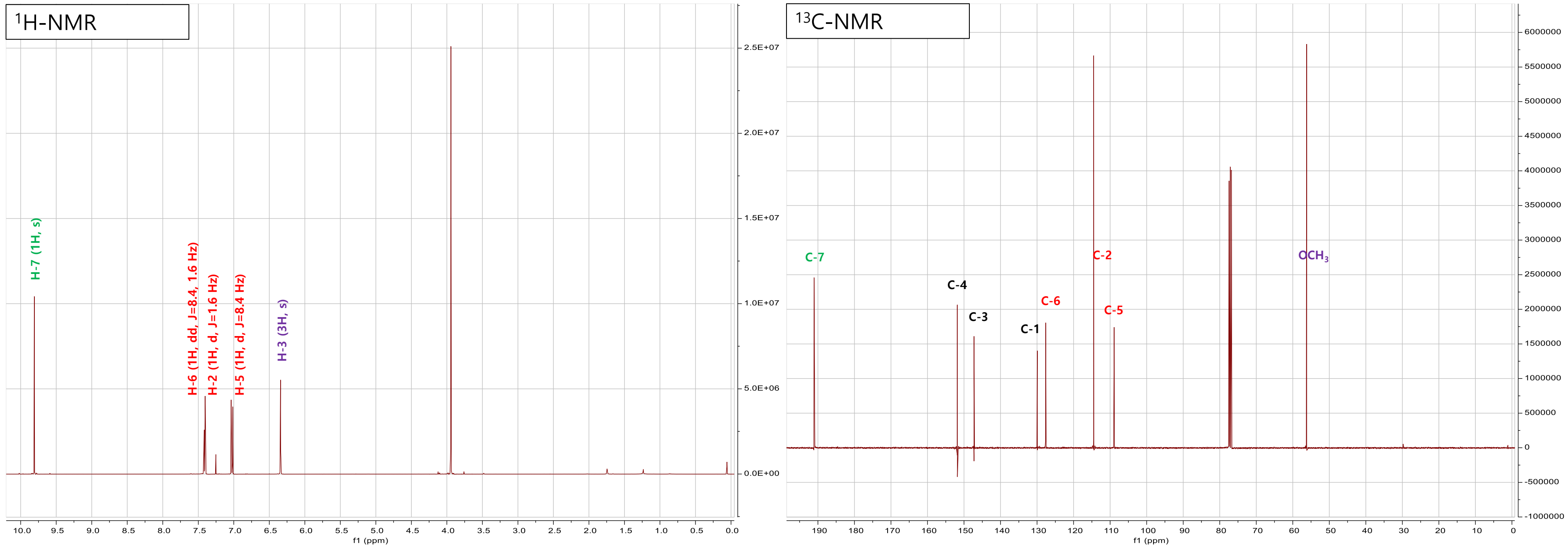

Figure S8. ^{1}H- and ^{13}C- NMR spectrum of compound **8**.

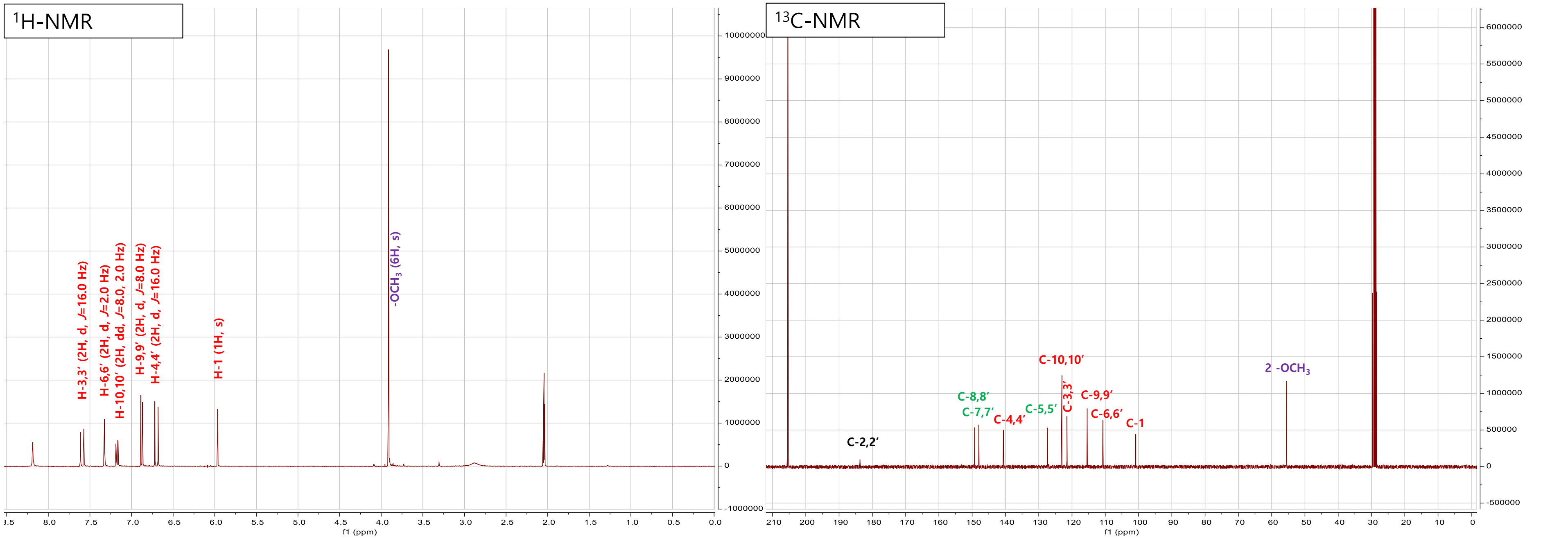

Figure S9. ^{1}H- and ^{13}C- NMR spectrum of compound **9**.

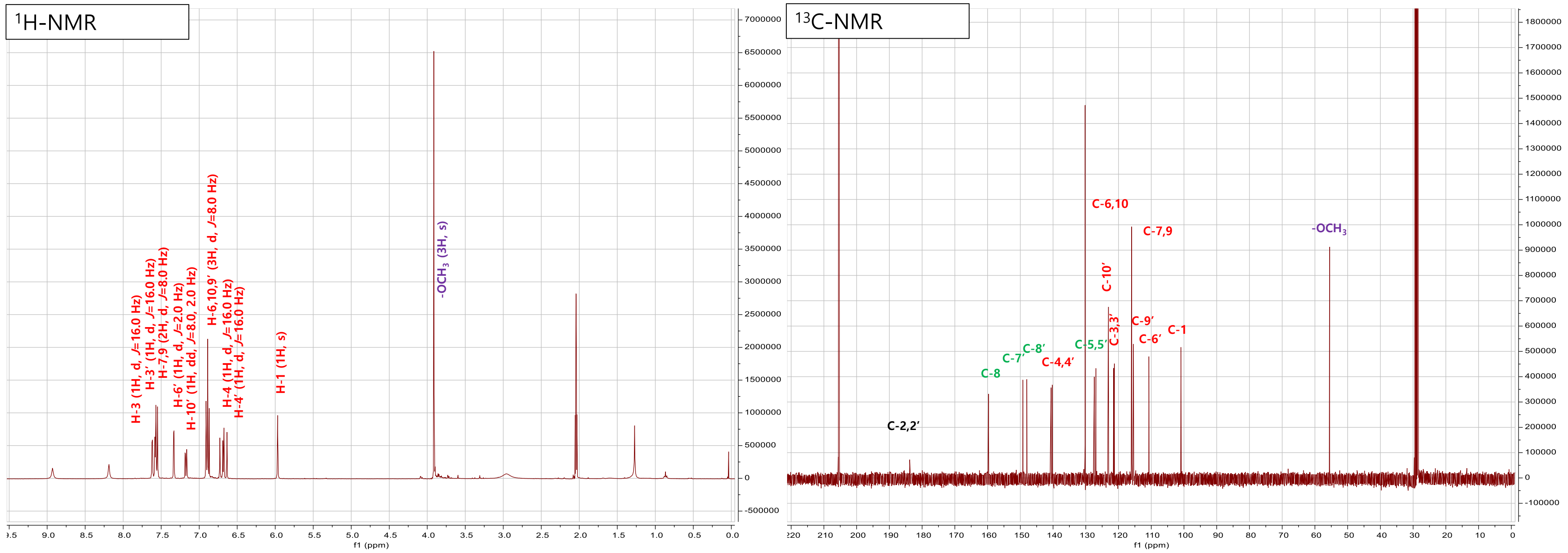

Figure S10. ¹H- and ¹³C- NMR spectrum of compound **10**.

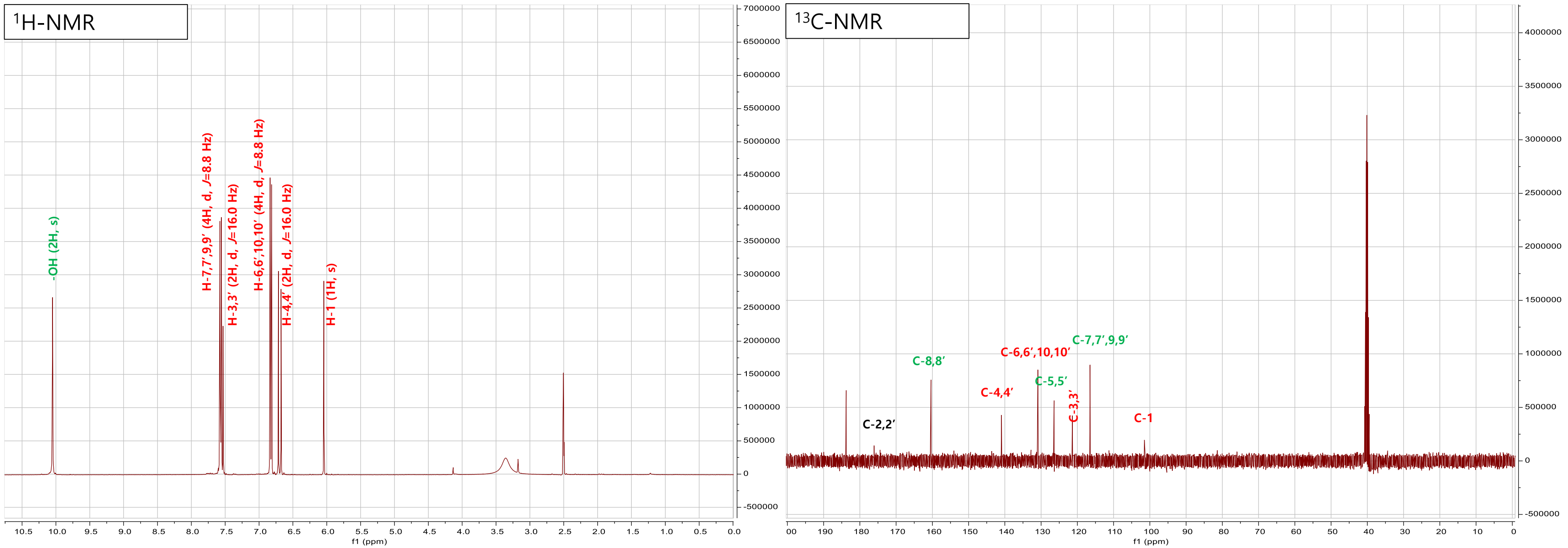

Figure S11. ^{1}H- and ^{13}C- NMR spectrum of compound **11**.

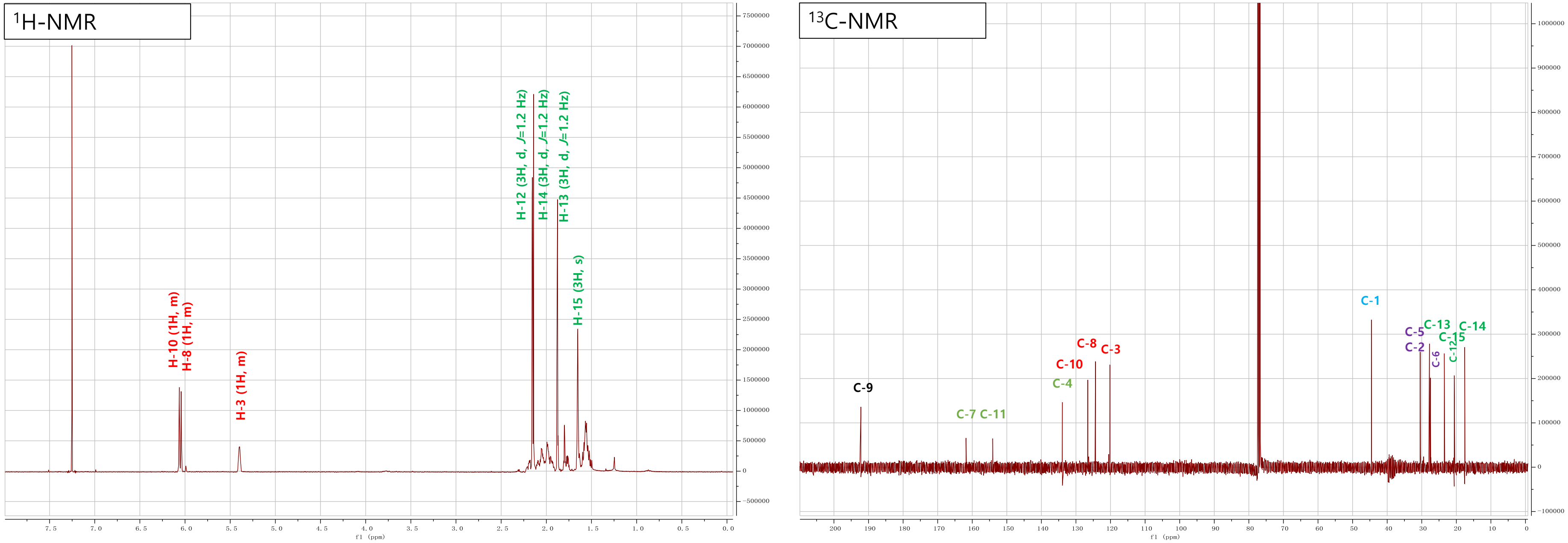

Figure S12. ^{1}H- and ^{13}C- NMR spectrum of compound **12**.

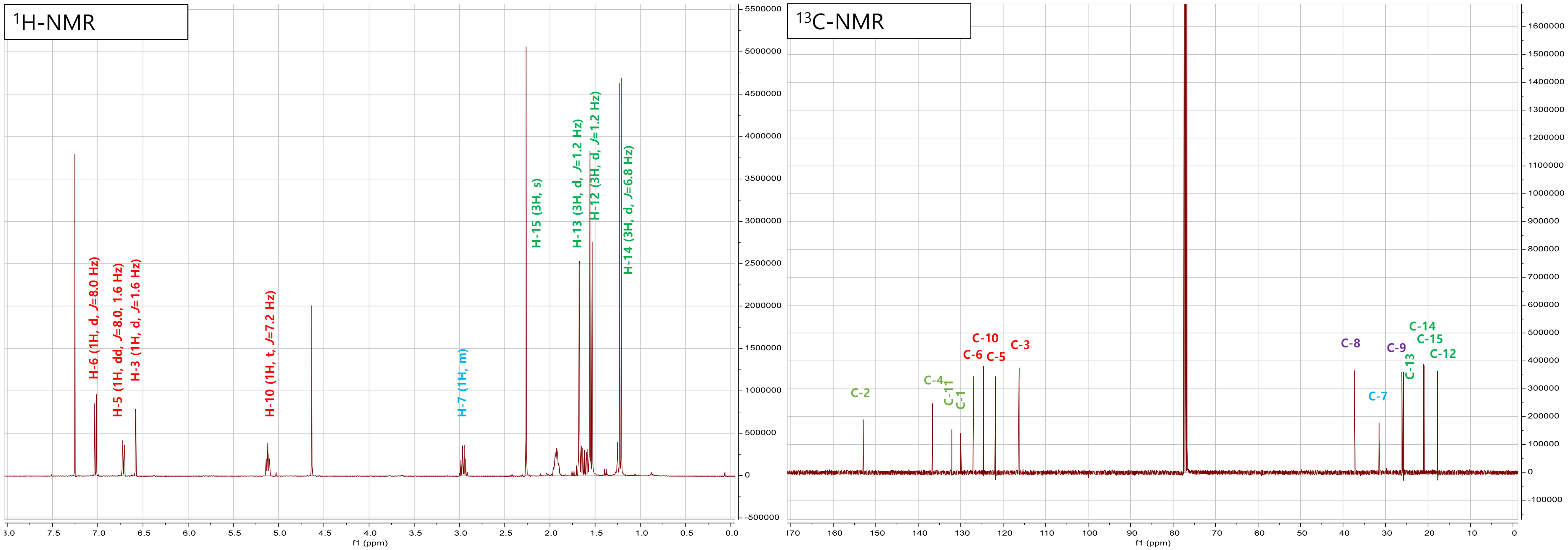

Figure S13. ^{1}H- and ^{13}C- NMR spectrum of compound **13**.

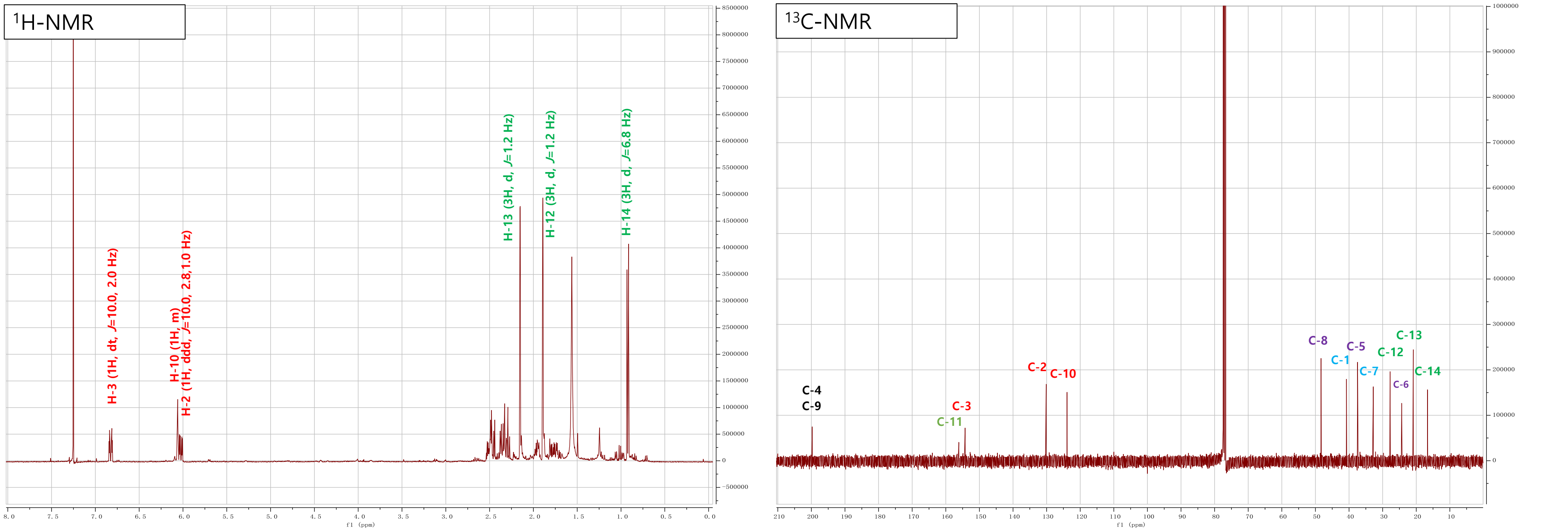

Figure S14. ^{1}H- and ^{13}C- NMR spectrum of compound **14**.

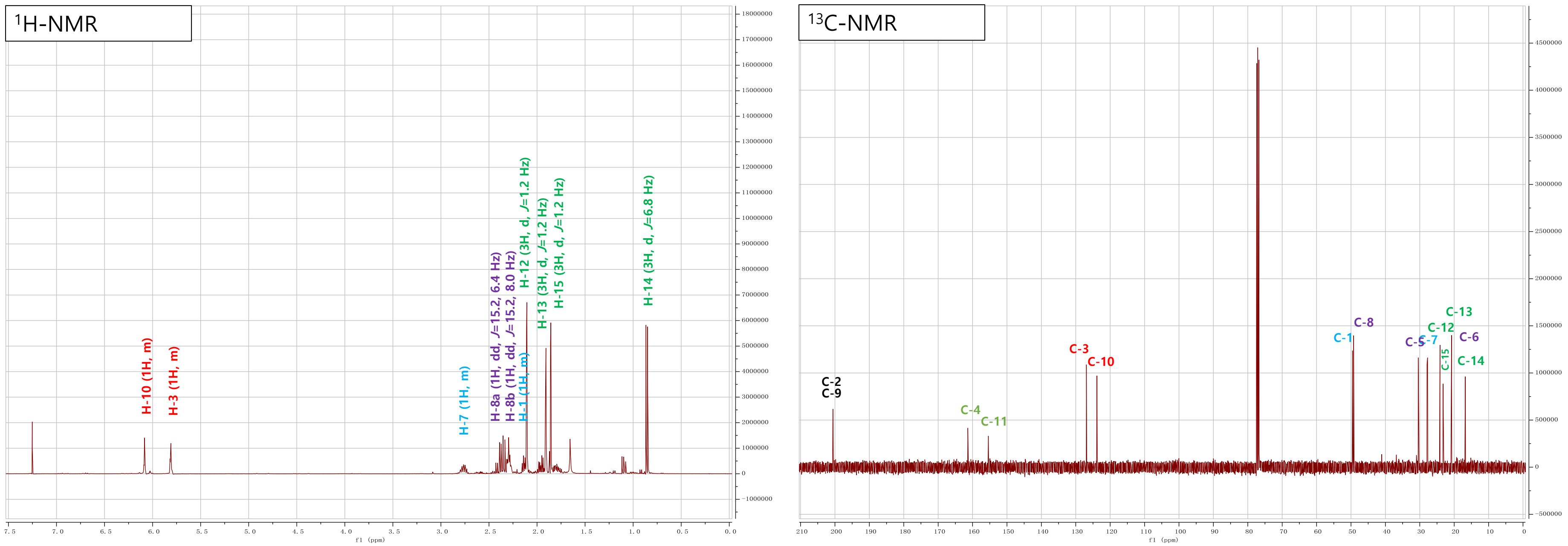

Figure S15. ^{1}H- and ^{13}C- NMR spectrum of compound **15**.

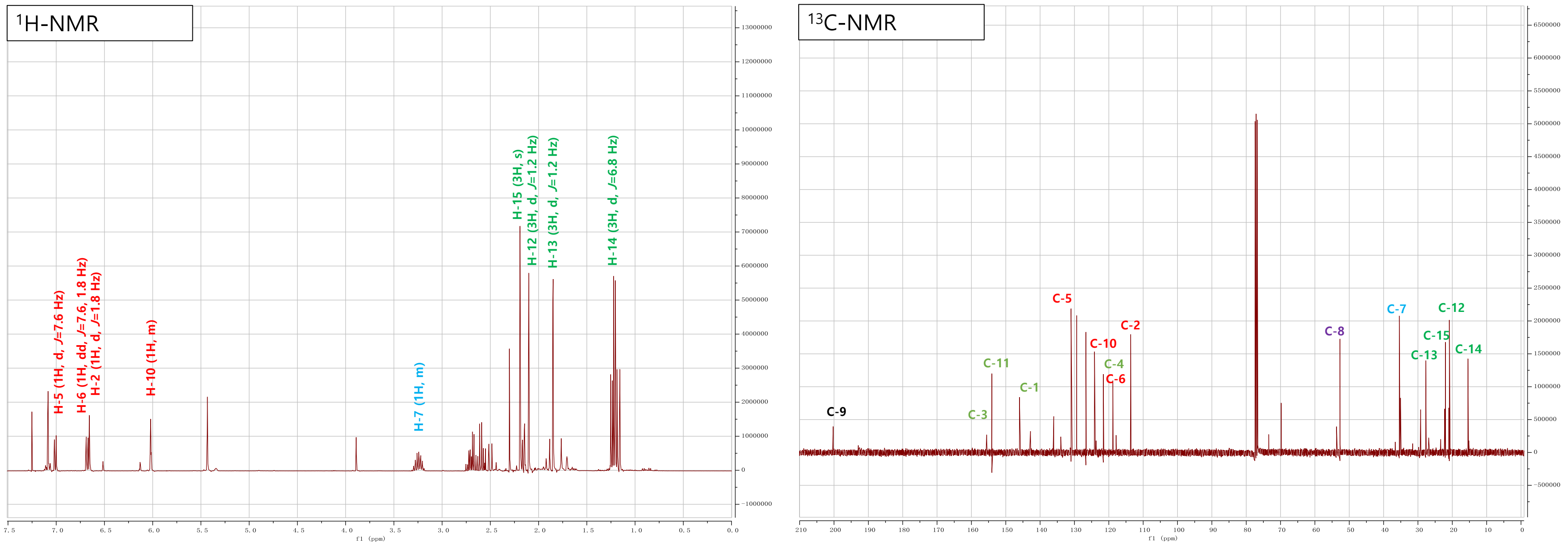

Figure S16. ^{1}H- and ^{13}C- NMR spectrum of compound **16**.

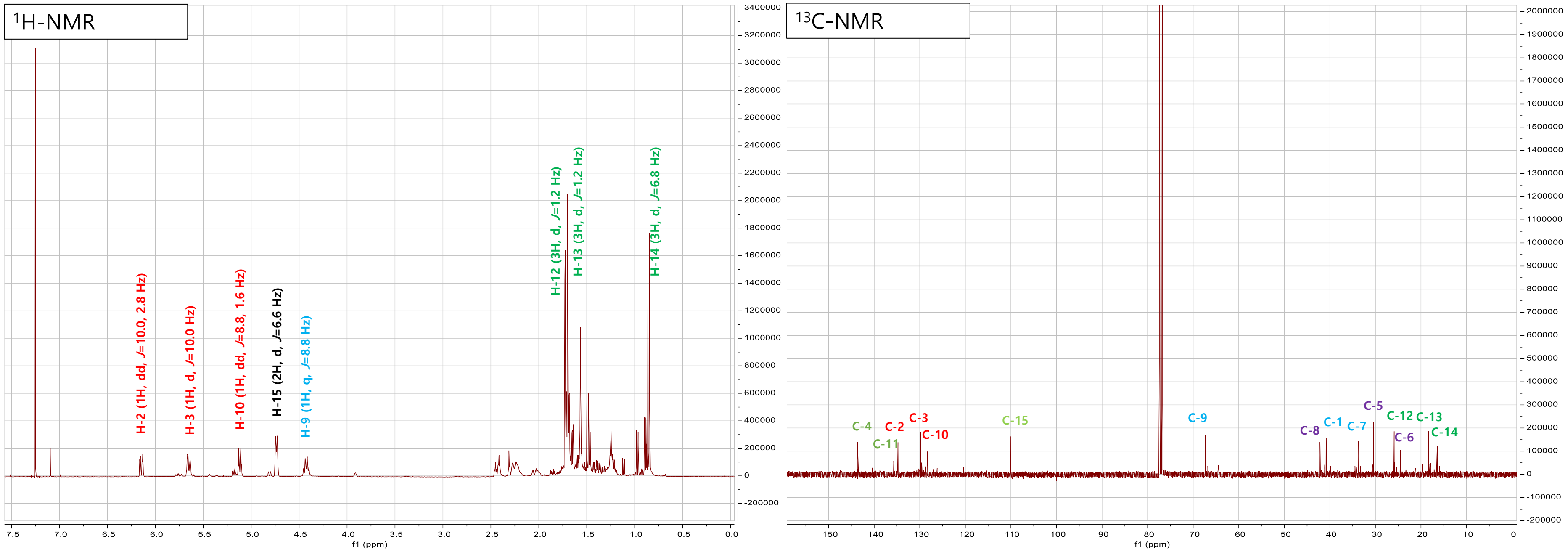

Figure S17. ^{1}H- and ^{13}C- NMR spectrum of compound **17**.
